# Comparative transcriptomic analysis reveals novel roles of transcription factors and hormones during the flowering induction and floral bud differentiation in sweet cherry trees (*Prunus avium* L. cv. Bing)

**DOI:** 10.1371/journal.pone.0230110

**Published:** 2020-03-12

**Authors:** Luis Villar, Ixia Lienqueo, Analía Llanes, Pamela Rojas, Jorge Perez, Francisco Correa, Boris Sagredo, Oscar Masciarelli, Virginia Luna, Rubén Almada

**Affiliations:** 1 Centro de Estudios Avanzados en Fruticultura (CEAF), Rengo, Chile; 2 Instituto de Investigaciones Agrobiotecnológicas (INIAB-CONICET), Consejo Nacional de Investigaciones Científicas y Técnicas (CONICET), Universidad Nacional de Río Cuarto, Río Cuarto, Córdoba, Argentina; 3 Instituto de Investigaciones Agropecuarias (INIA) CRI Rayentué, Rengo, Chile; Institute for Horticultural Plants, China Agricultural University, CHINA

## Abstract

In sweet cherry trees, flowering is commercially important because the flowers, after fertilization, will generate the fruits. In *P*. *avium*, the flowering induction and flower organogensis are the first developmental steps towards flower formation and they occur within specialized organs known as floral buds during the summer, nine months before blooming. During this period the number of floral buds per tree and the bud fruitfulness (number of flowers per bud) are stablished affecting the potential yield of orchards and the plant architecture. The floral bud development is sensitive to any type of stress and the hotter and drier summers will interfere with this process and are calling for new adapted cultivars. A better understanding of the underlying molecular and hormonal mechanisms would be of help, but unlike the model plant Arabidopsis, very little is known about floral induction in sweet cherry. To explore the molecular mechanism of floral bud differentiation, high-throughput RNA sequencing was used to detect differences in the gene expression of *P*. *avium* floral buds at five differentiation stages. We found 2,982 differentially expressed genes during floral bud development. We identified genes associated with floral initiation or floral organ identity that appear to be useful biomarkers of floral development and several transcription factor families (ERF, MYB, bHLH, MADS-box and NAC gene family) with novel potential roles during floral transition in this species. We analyzed in deep the MADS-box gene family and we shed light about their key role during floral bud and organs development in *P*. *avium*. Furthermore, the hormonal-related signatures in the gene regulatory networks and the dynamic changes of absicic acid, zeatin and indolacetic acid contents in buds suggest an important role for these hormones during floral bud differentiation in sweet cherry. These data provide a rich source of novel informacion for functional and evolutionary studies about floral bud development in sweet cherry and new tools for biotechnology and breeding.

## Introduction

Sweet cherry (*P*. *avium*) is perennial fruit tree that has a distinctive mode of reproductive development that differs significantly from Arabidopsis and annual species. In temperate regions, sweet cherry trees show a seasonal flowering that occurs over two growing season separated by a dormancy period. During the first growing season, at the end of the spring and during the summer, occurs the floral buds differentiation. The first sign of reproductive development is the meristematic apex acquiring a dome shape, which is followed by the formation of the flower primordia. These flower buds show between two and four small lateral protuberances, representing primordial bracts that subtend each flower primordium[1]. The sepal, petal, stamen and pistil primordial then differentiate in a centripetal way. Before leaf fall in autumn, the flower primordium appears as a dome with five sepal primordial and all whorls are distinguishable. Following dormancy, the final flower differentiation and blooming take place when temperature rise again in the spring[1,2]. Thus, the number of floral buds per tree and the bud fruitfulness (number of flower per bud) are established during first growing season. In sweet cherry (*P*. *avium*) trees, the flowering induction and many factors related to floral bud development (such as flower bud density, bud fruitfulness, double pistils and flower quality) are very important because they can influence the productivity and to determine in a considerable extent the commercial success of orchards[3]. These traits affect directly the potential crop yield as well as the plant architecture and several cultural practices (e.g. type of pruning employed)[4]. Variations in floral buds per tree, bud fruitfulness and flower development anomalies (double pistilated flowers) would be in part responsible for the inconsistent yields observed among years[5,6], converting the induction and initiation stage of flowering within the floral bud in a relevant process for stone fruit-industry interests.

In sweet cherry trees, the morphology of the apical meristems during floral bud initiation and differentiation has been well characterized[2]; however, until now, the molecular regulation of floral induction has not been thoroughly investigated. In contrast, the genetic, epigenetic, hormonal and environmental factors triggering the transition from the vegetative to the generative phase are best understood in the annual long-day (LD) *A*. *thaliana* plant, due to its generally acknowledged status as a model plant[7]. More than 90 genes were identified as regulating flowering time in *Arabidopsis*[8] and were grouped in different pathways that mediate and integrate environmental and endogenous signals. These pathways act as gene regulatory networks controlling floral transition and floral organ identification. Signaling pathways reacting to differential endogenous (autonomous, gibberellin, circadian clock, age) and environmental cues (vernalization, temperature, and photoperiod) converge on a small set of central flowering regulators, including *CONSTANS* (*CO*) and *FLOWERING LOCUS C* (*FLC*) transcription factor genes, which antagonistically regulate flowering[7,9]. FLC acts as a repressor of flowering and mediates the vernalization and autonomous pathways, whereas CO is a floral activator and mediates the photoperiodic pathway. Both genes regulate the expression of the downstream *floral pathway integrator* transcription factors *FLOWERING LOCUS T* (*FT*), *SUPPRESSOR OF OVEREXPRESSION OF CONSTANS 1* (*SOC1*), and *LEAFY* (*LFY*)[10]. FT interacts in the SAM with the bZIP transcription factor FLOWERING LOCUS D (FD) and this protein complex activates the *meristem identity genes LFY* and *APETALA1* (*AP1*) which irreversibly confer the transition from a vegetative to a floral meristem[7]. Then, the flower organs development take place and this differentiation process is mainly controlled by MADS-box transcription factors, known as floral organ identity genes (*AP1; AP3; PISTILLATA*, *PI; AGAMOUS*, *AG; SEPALLATA*, *SEP1-4*) which act together as transcriptional complexes (ABCE model) to ensure expression of the correct structural genes in each whorl of the developing flowers[11]. In addition, Arabidopsis flowering-related genes are regulated by phytohormones. Several studies demonstrated the importance of gibberellins as promoters of floral development. The exogenous application of GA_3_ promoted the formation of floral buds in *Arabidopsis* plants by activating family genes related to flowering in the apical meristems[12]. Others phytohormones, such as absicic acid, auxins and cytokinin have also been reported to play a role regulating the flowering network in Arabidopsis[13]. The cytokinin promotes the flowering through the activation of *Twin Sister of FT* (*TSF*)[14] whereas auxins are involved in flower primordium development through the regulation of *AUXIN RESPONSE FACTOR5/MONOPTEROS* (*ARF5/MP*) and *LFY* [15]. The ABA is regarded as a general repressor of flowering trough the transcription factors ABI4 and ABI5 that promote the transcription of the flowering repressor, *FLC*[16,17]. Exceptionally, ABA has a positive effect on flowering of *A*. *thaliana*, but it is restricted to extreme environments, known as the drought-escape response, in which plants accelerate flowering before dying[18]. As mentioned, much less is known about the hormonal and genetic regulation of flowering development in *Prunus* species[19]. Comparative and functional genomic approaches undertaken in some stone-fruit tree species are starting to provide information on the conservation of flowering genes between *Prunus* species and herbaceous plant species[19,20]. Genes with homology to the photoperiod pathway (*CONSTANS*, *CO*)[21], floral integrator-like (*FT* and *SOC1*)[22,23], meristem identity-like (*LFY*, *AP1*, *AP2* and *MADS-box*)[20] and floral organ identity-like genes have been found in *Prunus spp*. species[19,24] suggesting that these genes could play important roles in the transition from vegetative to the reproductive phase in sweet cherry trees. Although some genes with homology to Arabidopsis genes involved in flowering induction, flower evocation and differentiation have been found in *P*. *avium*, the reduce number of flowering-related genes reported in stone-fruit tree species suggest that a “plethora” of genes potentially involved in flowering regulation in *Prunus* species are waiting to be discovered.

In order to discover genes potentially involved in flowering induction and flower development in *P*. *avium*, we surveyed genome-wide expression patterns by RNA-seq in early stages of floral bud development and vegetative tissues (leaves and roots). We identified homologs of a number of well-known genes associated with floral initiation or floral organ identity that appear to be useful biomarkers of floral development and several transcription factors with novel potential roles during floral transition. We analyzed in deep the transcriptional patterns of several members of the MADS-box gene family of transcription factors. Furthermore, we found hormonal-related signatures, mainly associated to abscisic acid, auxins and citokinins, in the floral bud transcriptomes and we evaluated the dynamic changes of absicic acid (ABA), zeatin (Z) and indolacetic acid (IAA) levels in floral buds and bud-adjacent leaves in order to determinate their potential role during floral transitions in sweet cherry. To the best of our knowledge, this is the first work that provides an in-depth study of the transcription factors and hormones patterns during the flowering induction and flower bud differentiation in *P*. *avium* cv. Bing, in order to indentify key genes in this economically important tree species.

## Materials and methods

### Plant material and sample collection

Floral buds and vegetative organs (e.g. leaves) were sampled from 9-yr-old grafted clonal *Prunus avium* cv. Bing trees grown in an orchard in O´higgins Region (S34°19’16.8”, W70°50’02.2”), Chile. The owner of the land, Instituto de Investigaciones Agropecuarias (INIA), gave us permission to conduct the study on the sweet cherry orchard because several of authors of this research article belong to INIA. Tissues were sampled between December 2015 and March 2016, with four sampling dates (S1: December 16; S2: January 15; S3: February 15; S4: March 15). Also, floral buds were sampled during dormancy (D) in June 2016. For every floral bud stage, one hundred thirty floral buds were sampled from at least six trees (biological replicates) and pooled. Forty S1-adjacent leaves were sampled from at least six trees (biological replicates) and pooled. One hundred flowers were sampled during anthesis from at least six trees (biological replicates) and pooled. Flowers were carefully separated in pistils, anthers, carpels and petals and frozen individually. Also, eighty fruits in two developmental stages were collected (IF, inmature and MF, mature or harvest time fruits). The reproductive growth stages analyzed are in agreement with the extended BBCH’s scale[1] and are the following: Flower whorls were collected from flowers at anthesis stage 65, IF stage 77 and MF stage 89. Roots from a F12 rootstock (*P*. *avium*) were also sampled from at least six trees (biological replicates) and pooled. All collected samples were immediately frozen in liquid nitrogen and then stored at -80°C. Sampling was performed at approximately 10 a.m. to reduce the possibility of differences in gene expression due to circadian oscillation, as the plants were grown under field conditions.

### Floral tissue microscopy

Samples of floral buds were fixed in FAA (formaldehyde–acetic acid–ethanol), and dehydrated in ethanol series (15 min in each of 70%, 80%, 90%; 30 min in 100% and a further 60 min in 100%). Samples were subsequently cleared at 37°C with mixtures of ethanol:Histo-clear (National Diagnostics, Georgia, USA)(3:1; 1:1; 1:3 for 1h) and then, in pure Histo-clear (30 min and over-night). Samples were embbebed at 54°C in histo-clear:paraffin (Histoplast LP, Thermo Scientific, Michigan, USA)(1h in 3:1; 1:1; 1:3 and pure paraffin overnight). Specimens were transferred to plastic molds, covered with fresh wax at 54°C, then polymerized by cooling at room temperature. Paraffin blocks containing the floral buds were sectioned with a Micro Rotary microtome HM 325 (Thermo Scientific, Walldorf, Germany). Sections were mounted on glass slides and viewed in a microscope (Olympus BX53). Images were obtained with a MicroPublisher camera (QImaging, BC, Canada).

### Gene expression analysis

Total RNA was isolated from 3 gr. of floral buds, roots or leaves. Total RNA integrity was verified by formaldehyde agarose gel electrophoresis and RNA purity was assessed by OD260/OD280 nm absorbance ratios. Following DNase treatment of total RNA, first-strand cDNA synthesis was carried out with oligo (dT) and 2 μg of total RNA for all samples, following the manufacturer’s instructions (Thermoscript RT-PCR System, Invitrogen). Gene expression levels were determined by quantitative PCR (qPCR) using the Mx3000P QPCR System (Agilent Technologies, USA). All reactions were performed with Brilliant SYBR Green Master Mix (Stratagene, USA) following the manufacturer’s instructions. Three high quality total RNAs (biological replicates) were obtained for each plant sample and used for genes expression analysis by qPCR[25]. For every biological replicate, qRT-PCR reactions were run in duplicate (technical replicates) with 10 μl Master Mix, 0.5 μl 250 nM of every primer, 1 μl diluted cDNA, and nuclease-free water to reach a final volume of 20 μl. Controls (without cDNA and RNA without RT) were included in all runs. Fluorescence was measured at the end of each amplification cycle. Amplification was followed by a melting curve analysis with continual fluorescence data acquired during the 65–95°C melt. Expression was normalized against the *Prunus* Translation Elongation Factor 2 gene (*PpTEF2*, GenBank Database accession number TC3544) due to its consistent transcript level in the tissues[25]. The primers used for gene expression analysis are described in **S4 Table.**

### Construction of cDNA library and Illumina HiSeq 4000 sequencing

RNA samples, previously treated with DNAse I, were sent to Genoma Mayor Company (Santiago, Chile) for mRNA-seq library construction and sequencing using Illumina HiSeq 4000 (Illumina, Inc., San Diego, CA, USA) next-generation platform technology. For mRNA-seq library construction from total RNA, an mRNA-seq sample preparation kit (TruSeq Stranded mRNA^™^, Illumina Inc., San Diego, CA, USA) was used in accordance with the manufacturer’s instructions. Briefly, the total RNA was treated with DNase I and poly-T oligo attached magnetic beads to elute poly-(A+) mRNA. The purified mRNA was fragmented prior to cDNA synthesis. The cleaved mRNA fragments were primed using random-hexamer primers and reverse transcriptase (Invitrogen, Carlsbad, CA, USA) to synthesize first-strand cDNA. The synthesis of second-strand cDNA was accomplished using RNase H (Invitrogen) and DNA polymerase I (New England BioLabs, Ipswich, MA, USA). Subsequently, end-repair of double-stranded cDNA was performed using T4 DNA polymerase, the Klenow fragment, and T4 polynucleotide kinase (New England BioLabs). The end-repaired cDNA was ligated to Illumina paired-end (PE) adapter Oligo-mix with T4 DNA ligase (New England BioLabs) at room temperature for 15 min. Suitable fragments were then sequenced in a PE (paired-end) pattern on an Illumina sequencing machine. The sequencing data received from the sequencer were transformed by base calling into raw reads and stored in fastq format.

### Bioinformatics analysis of transcriptomic sequences: Assembly, abundance and annotation

Twenty one mRNA samples were sequenced to obtain about ~ 29 × 10^6^ paired-end (PE; 100 bp) reads using Illumina HiSeq 4000 in average in each sample (totally ~ 617 x 10^6^ PE reads). All read pairs were quality checked for potential sequencing issues and contaminants using FastQC 0.11.7 (http://www.bioinformatics.babraham.ac.uk/projects/fastqc), Adapter sequences, primers, Ns, and reads with quality score below 30 were trimmed using AfterQC[26](**S3 Table**). Reads with a remaining length of fewer than 35bp after trimming were discarded. These processed reads were assembled into a *de novo* transcriptome using Trinity v2.6.5 software[27]. For the assembly, reads were pooled from all libraries and the minimum sequence length was set to 200. Trinity was run with the following parameters: library normalization with maximum read coverage 50, and FR strand specific read orientation. The program cd-hit-est with a sequence identity threshold of 0.95 was used to reduce the redundancy of the assembly. Raw reads were mapped against the *de novo* transcriptome using Bowtie2 software. Relative abundance for each transcript and unigene were estimated by aligning the reads to the transcriptome assembly using RSEM v1.3.0. Best scoring open reading frames were extracted from Trinity transcripts and translated into protein sequences using TransDecoder from the Trinity software package. For the identification of differentially expressed genes (DEGs) we used the R/Bioconductor package DESeq2 1.12.4 (p-value at most 1e-3 and at least 4 fold-change). The identification of significantly overrepresented annotations in DEGs was performed using the analysis tool provided by AgriGO V2.0[28]. The DEGS for each transition (S1-S2, S2-S3 and S3-S4) were imported into AgriGO’s Singular Enrichment Analysis tool using the *Prunus persica* probe identification number with the peach gene model chosen as background; all other options were left at their default setting. Annotation groups with P<0.05 were identified as significantly overrepresented.

### Annotation and characterization of the *de novo* transcripts

Annotation for all the unique transcripts (>200 bp) was done using BLASTx homology search against NCBI Refseq Plants protein Database, NCBI-nr database and Genome Database for Rosaceae (GDR). BLAST hits with e-value scores ≤0.001 and query coverage above 60% was considered as homologous proteins and AWK script was used for filtering reciprocal best hits. GO term enrichment analysis was conducted on annotated transcripts present in the different clusters employing GO and COG databases.

### Characterization and phylogenetic analysis of MADS-box proteins in sweet cherry

An extensive search to identify MADS domain-containing proteins in the sweet cherry genome was performed using a Hidden Markov Model (HMM), for the MADS-box (SRF-TF PF00319) and K-box domains (K-box PF01486). As well, MADS-box proteins in sweet cherry proteome (*P*. *avium* V1.0) were searched for using the BLASTP algorithm. The existence of the conserved MADS-box motif was confirmed in each case using conserved domain analysis (http://www.ncbi.nlm.nih.gov/Structure/cdd/wrpsb.cgi). Finally, sweet cherry MADS-box candidate proteins were manually confirmed using MEGA v7.0. The sequences lacking MADS domains were rejected in the following analysis. Based on the InterPro results, the candidate MADS-box genes were classified as type I or type II according to whether they contained K domains. Within each type, we assigned a uniform name to every gene according to the order of their gene IDs. The deduced amino acid sequences from *Arabidopsis* and *P*. *mume* MADS-box proteins were obtained from Vining et al. (2015)[29] and Xu et al. (2014)[30]. ClustalX was used to create multiple alignments. The resulting alignment was used to assemble the phylogenetic tree by the neighbor-joining method with MEGA v7.0.

### Determination of phytohormone levels

At four floral bud developmental stages (S1, S2, S3 and S4) and S1 bud-adjacent leaves, 150 mg dry weight of buds and leaves were used for determination of phytohormone levels. For abscisic acid (ABA), auxins (indol acetic acid, IAA) and citokinis (zeatin, Z) analyses, lyophilized plant material (buds and leaves) was weighed and extracted in 5 ml of extraction buffer at pH 2.8–3. 50 ng of 2H6-ABA, 2H5-trans-ZEATIN and 2H5-AIA) (OlChemIm Ltd, Olomouc, Czech Republic) were added as internal standards. Extracts were transferred to 50 ml tubes, centrifuged at 8000 rpm for 15 min, and supernatants were collected and mixed with ethyl acetate. Then, the organic phase was extracted and evaporated at 37 ºC. Dried extracts were dissolved in 1500 μl methanol and evaporated and immediately resuspended in 50 μl pure methanol. 10 μl of each sample were injected onto a Liquid Chromatograph (LC) with Electrospray Ionization (ESI) (Waters Corp., New York, NY, USA).

### Liquid chromatography

Analyses were performed using an Alliance 2695 (LC Separation Module, Waters, USA) quaternary pump equipped with auto-sampler. A Restek C18 (Restek, USA) column (2.19 x100 mm, 5 μm) was used at 28ºC, with injected volume 10 μL. The binary solvent system used for elution gradient consisted of 0.2% acetic acid in LC-MS grade water (solvent B), and methanol (100%) (solvent A), at a constant flow-rate of 200 μL min-1. A linear gradient profile with the following proportions (v/v) of solvent A was applied [t (min), % A]: (0, 40), (25, 80), with 7 min for re-equilibration.

### MS/MS experiments

MS/MS experiments were performed on a Micromass Quatro UltimaTM PT double quadrupole mass spectrometer (Micromass, Manchester City, UK). All analyses were performed using turbo ion spray source (ESI) in negative ion mode with the following settings for gibberellins: capillary voltage -3250 V, energy cone 35 V, RF Lens1 (20), RF Lens2 (0.3), source temp. 100º C, desolvation temp. 350 ºC, gas cone 100 L h-1, gas desolvation 701 L h-1, collision cell potential of 15 V and multiplier (650). MS/MS parameters were optimized in infusion experiments using individual standard solutions of each hormone. MS/MS product ions were produced by collision-activated dissociation of selected precursor ions in the collision cell of the double quadrupole mass spectrometer, and mass was analyzed using the second analyzer of the instrument. In negative mode, the spectrum for each hormone gave deprotonated molecule [M–H]. Quantitation was performed by injection of samples in multiple reaction monitoring (MRM) modes, since many compounds could present the same nominal molecular mass. The combination of parent mass and unique fragment ions was used to selectively monitor hormones. MRM acquisition was performed by monitoring the 263/153 and 269/159 for ABA and (2H6)-ABA; 174/130 and 179/135 for IAA and (2H5)-IAA; and 118/135 and 223/135 for Z and (2H5)-trans-zeatin respectively, with dwell 2000 ms for each transition. Data were acquired and analyzed using MassLynxTM 4.1 and QuanLynxTM 4.1 (Micromass, Manchester, UK) software. For quantification, values were obtained from a calibration curve previously constructed using known amounts of each hormone and their pure standard (Sigma, St. Louis, MO, USA)/ deuterated internal standard ratio.

**PCA, k-means clustering, heat map and Venn diagrams.** Principal component analysis (PCA), k-means clustering and heat maps were performed using the Genesis 1.8.1 software[31]. A Venn diagram of distribution of DEGs was constructed using the online Venny tool (http://bioinfogp.cnb.csic.es/tools/venny/).

### Statistical analysis

For qPCR results and hormone levels, a one-way analysis of variance with Tukey-Kramer multiple comparison test with a confidence level of 95% was performed using SigmaPlot software, version 12.

## Results

### Floral bud development during floral transition

**Fig 1:**
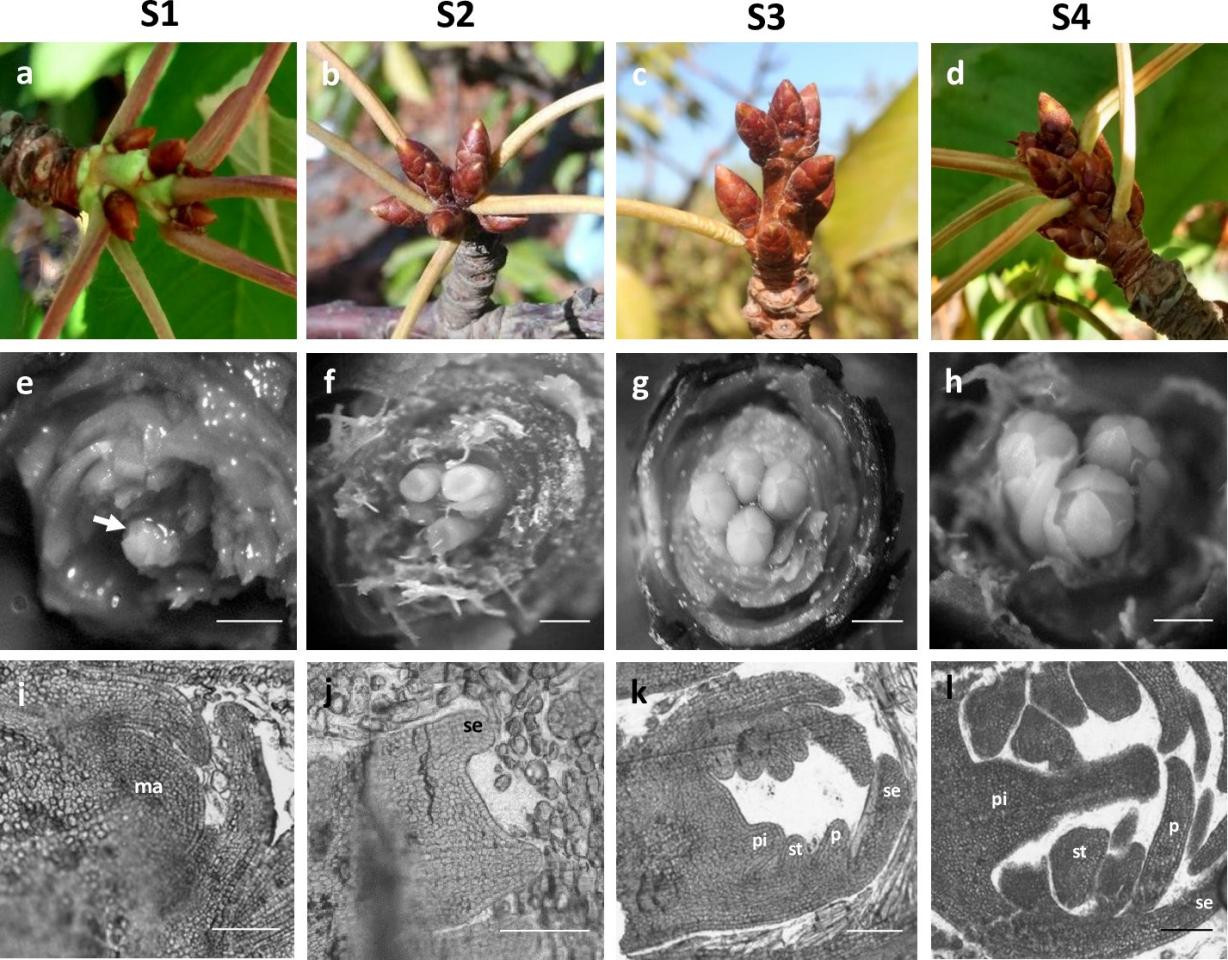
Floral buds sampled for RNA-seq analysis. a,e,i: Stage 1 (S1), rounded meristem; b, f, j: S2, flower primordia; c, g, k: S3, early differentiation of the flower whorls; d, h, l: S4, differentiation of the pistils (Bars: 0,5 mm in e-h, 100 μm in i-l). Abbreviations: ma, meristem apex; se, sepals; p, petals; st, stamens; pi, pistil.

Before the transcriptome study, we analyzed the developmental process of floral buds in a morphological analysis. The flowering induction was evidenced within the floral buds by a meristematic apex with a dome-like shape and we termed this time point as S1 stage (S1) (**Fig 1A, 1E and 1I**). The S1 stage was observed at the end of the spring (Dec. 16 in the southern hemisphere). Then, the flower primordias differentiate during the summer (Jan. 15) and they appeared as a cup-like structure with 5 sepal primordias in their margin (**Fig 1B, 1F and 1J**). We termed this time point as S2 stage (S2). Forward in the development, the flower primordium appears as a dome with five sepal and within it, the floral whorls continued their development and the petal, stamen and pistil primordias were evidenced (**Fig 1C, 1G and 1K**) (Feb. 15). We termed this time point as S3 stage. At the end of summer (March 15), the flower primordium appears as a dome with five sepal and all whorls are distinguishable (**Fig 1D, 1H and 1I**). We termed this time point as Stage S4 (S4). Thus, based on morphology, we considered four flowering related stages (S1-S4 stages) and used them for high-throughput RNA-seq study of floral transition.

Growth kinetic during flower induction was studied in sweet cherry buds. The bud length increased >100% between the S1 and S4 stages during flowering induction and development of flower whorls, but only ~4% during dormancy (April-June, D stage) (**S1A–S1C Fig**). The bud width increased almost 60% between the S1 and S4 stages during flowering induction and development of flower whorls, but little changes during dormancy (April-June, D stage) were observed (**S1B–S1D Fig**).

### Global analysis of RNA-seq data

To obtain a genome-wide view of the transcriptome changes of the floral transition in *P*. *avium* cv. Bing, four developmental stages (S1-S4) of floral buds were constructed using RNA-seq with three biological replicates performed for each stage. Also, we include the transcriptome of floral buds at dormancy (D stage), which was collected during the winter (June 15, Southern hemisphere), in order to compare the transcriptomes of actively growing S1-S4 buds with the transcriptome of D buds in which the floral organogenesis seems to be stopped. For the functional annotation of genes required for flowering induction in sweet cherry, we compared the transcriptome of floral buds at S1 stage (flowering induction) with the transcriptomes from vegetative tissues such as S1-bud adjacent leaves and roots. A total of 616,862,772 raw reads were obtained during transcriptomic sequencing. The raw data were uploaded to NCBI under the accession numbers BioProject Database (ID PRJNA529895). After stringent quality checks and data trimming, a total of 613,460,281 high quality reads (>Q30) were obtained. In total, 113,878 unigenes were produced after de novo assembly by Trinity (**S1 Table**). After applying cd-hit-est to reduce the redundancy of the assembly, the number of contigs decreased to 113,829. The average length of all transcripts was 790 bp, and N50 (length for which half of the total bases are in contigs of this length or longer) was 1,750 bp. To reduce the number of potential spurious contigs or to filter out transcripts that belong to non-plant species (e.g. endophytic fungi), we filtered them based on the homology with proteins in Refseq database (using BLAST-x), the orthology with proteins from *Viridiplantae* (using eggNOG) and/or the presence of candidate coding region (using TransDecoder, we selected transcripts with ORFs that are translated in at least 100 amino acids long). We found that 81,376 unigenes showed homology with proteins described in the Refseq database. The total number of protein coding transcripts among the final non-redundant transcripts was 44,130. The eggNOG mapper revealed orthology with *Viridiplantae* for 26,598 of sequences analyzed. The number of final non-redundant unigenes considered for downstream analysis such as functional annotation, abundance quantification and differential expression analysis was 27,429.

### Differential expression among floral buds and vegetative tissues

**Fig 2:**
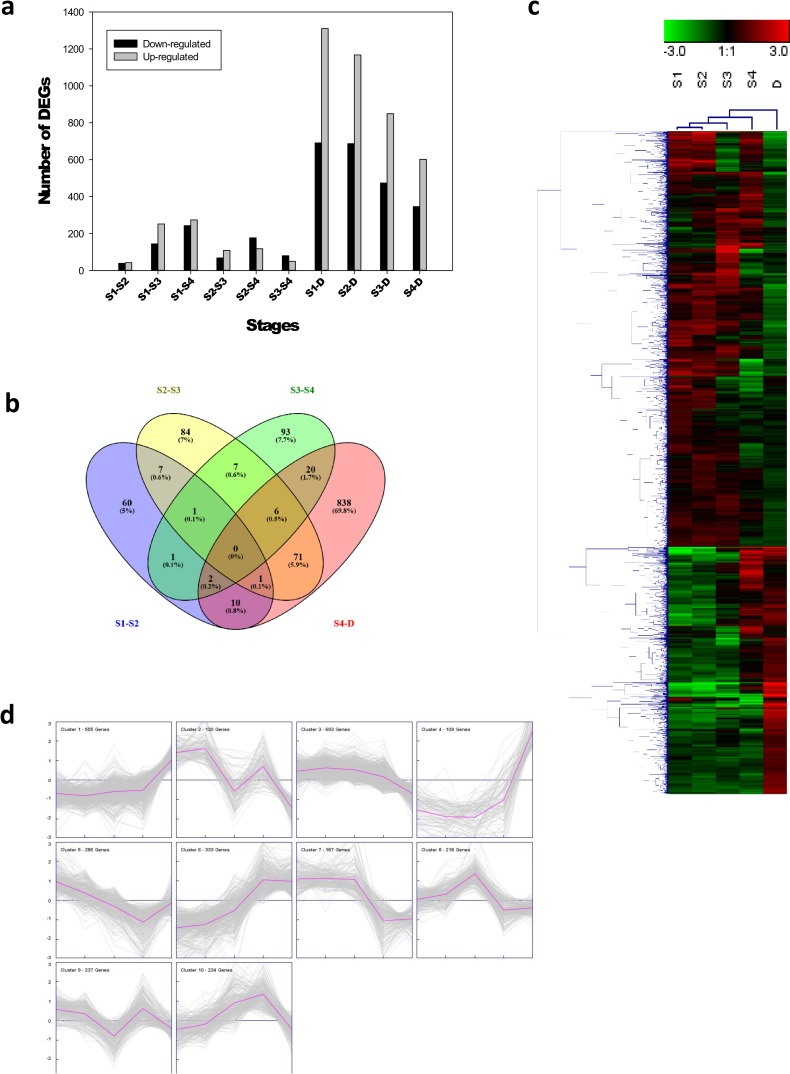
Gene expression dynamics during the different stages of floral bud development in *P*. *avium*. a) The number of up- and down-regulated genes during the various stages of floral buds (S1-S4) development with respect to the preceding stage or dormancy (D). b) Venn diagram of DEGs in the five (S1-S4 and D) transcriptome datasets. c) Hierarchical cluster analysis of 2,982 DEGs. The heat maps represent the log2 fold changes (FDR≤0.05) of DEGs. Red and Green colors represent up- and down-regulated genes, respectively. Scale, representing the signal values, is shown at the top of the Fig d) K-means clustering of the DEGs during the five developmental stages. Black numbers on the top are the number of genes for each cluster.

To identify differentially expressed genes (DEGs), we compared the transcriptomes of floral buds during flowering induction (S1), flower whorls differentiation (S2-S4) and dormancy (D stage). The Principal Components Analysis (PCA) and Replicate Correlation (RC) analysis of RNA-seq data showed that two out the three biological replicates from the S1, S2 and S3 samples were clustered whereas the third biological replicate was distantly positioned (see PCA and RC plots in supplementary information available in Figshare Project N°69965). Therefore, we used the two more closely related biological replicates from each floral bud stage (S1-S4, D) to identify the DEGs. In total, 2,982 DEGs were found to be significantly changed during floral transition (S1-S4 and D). In successive pairwise comparisons of floral buds, we found 82 DEGs between S1-S2, of which 43 were up-regulated and 39 were down-regulated. Between S2-S3, there were 177 DEGs, with 109 up-regulated and 68 down-regulated. Between S3-S4, we found 130 DEGs, of which 50 were up-regulated and 80 were down-regulated. The largest numbers of DEGs were found when S1-S4 was compared with floral bud in dormancy (D) (**Fig 2**). Furthermore, we compared the overlap of pairwise comparison regarding the identity of DEGs by a Venn diagram (**Fig 2**). A large proportion of DEGs exhibited developmental stage-specific differential expression, and no one gene was found to be common in all the comparisons. Principal component analysis (PCA) carried out on the whole-gene expression data set revealed the distinctness of S1-S4 from the D buds (**S2 Fig**), suggesting the presence of different transcriptional programmes.

The transcriptomes of floral buds at S1 and *P*. *avium* vegetative tissues (leaves and roots) were compared in order to find genes preferentially expressed in floral buds during flowering induction. We found 4,609 and 5,010 DEGs between S1 floral buds/S1-adjcent leaves and S1 floral buds/roots, respectively.

The annotated DEGs were classified into three GO categories namely *biological process*, *molecular function* and *cellular component*. Without considering the direction of change of the individual transcripts, the top enriched GO terms among the three comparisons (S1-S2, S2-S3 and S3-S4) are shown in **S3 Fig**. Under *cellular components*, majority of DEGs were associated with membrane, membrane part, cell, cell part and organelle (**S3 Fig**). The *molecular functions* belonged to “binding”, “structural molecule activity”, “transporter activity”, “catalitic activity” and “nucleic acid binding transcription factor activity”. The number of genes mapped to “binding” GO were higher in S3-S4 as compared to S1-S2 or S2-S3 transitions (**S3 Fig**). Also there was a higher number of genes asociated to “transport activity” in S1-S2 than in S2-S3 and S3-S4 transitions. Outstanding, the number of genes in the “nucleic acid binding transcription factor activity” GO was a higher in S1-S2 and S2-S3 transitions than in S3-S4 transition. The DEGs were enriched in *biological processes* related to the regulation of biological, cellular, metabolic and single-organism processes. The significant enrichment analysis identified several GO terms viz. responses to stimulus and signalling process were overrepresented in DEGs from S2-S3 and S3-S4 transitions (**S3 Fig**).

### Transcription factors

Understanding the role of transcription factors (TFs) is essential in reconstructing developmental regulatory networks. Therefore, we scutrinized the DEGs from floral buds in S1-S4 and D developmental stages in order to find transcription factors that could play a role during the flowering transition in sweet cherry. We found 206 genes codifyng TFs and representing 34 out the 58 TFs families examined[32]. The most represented TFs families in the DEGs were the *ERF*, *MYB* and *MADS-box* family with 16%, 15% and 9% of differentially expressed TF transcripts, respectively. Other families such as *NAC*, *WRKY* and *bHLH* were represented by around a 6% of differentially expressed TF genes. Hierarchical clustering of the set 206 TFs revealed 108 TFs that were highly expressed in S1-S4 floral buds whereas 25 TFs were mainly expressed in dormant (D) buds (e.g. *bHLH*, *NAC* and *MYB* gene families expression patterns in S4 Fig). Furthermore, 73 TF genes were concomitantly expressed in S1/S4-D buds. We scrutinized the DEGs in S1 buds, S1-adjacent leaves and roots in order to find TFs preferentially expressed in floral buds. We found 487 DEGs codifying TFs and clustering analysis revealed that 138 TFs genes were mainly expressed in S1 buds. The most representative TFs families were *ERF*, *MYB* and *bHLH* with 11%, 7% and 6% of DEGs codifying TFs, respectively. The analysis of expression patterns revealed that *MADS-box* genes and several other TFs family members seems to be involved in the differentiation of floral buds (S1-S4) in this species and they emerged as good candidates genes for further analysis.

### *MADS*-box genes in the sweet cherry genome

In plants, *MADS*-box genes are master regulators of developmental processes [47]. Eighteen MADS-box genes were differentially expressed during the floral bud development highlighting the importance of these transcription factors during the sweet cherry floral bud development. In order to determine to which subfamilies belonged the differentially expressed MADS-box genes, we analyzed the genomic organization of this gene family in *P*. *avium* using the genome information available[33]. As a result of an extensive search for MADS-domains containing proteins in the sweet cherry genome through profile hidden Markov models (HMMER), 78 distinct putative *MADS*-box transcription factors were identified in *P*. *avium*. The conserved MADS-box and K domains were further analyzed using conserved domain analysis tools (http://www.ncbi.nlm.nih.gov/Structure/cdd/wrpsb.cgi)[34] and subsequently, 75 genes were confirmed as sweet cherry *MADS-box* genes (**S2 Table**). Four *P*. *avium MADS-box* genes (XP_021800168.1, XP_021808124.1, XP_021830264, XP_021833461) encode putative truncated proteins since only conserve the K-domain and were not included in our analysis. The domain analysis also revealed that 33 genes possessed the MADS-box and K domains; therefore, they were classified as type II (MIKC type) MADS-box genes, and 38 genes were confirmed as type I (including Mα, Mβ, Mγ and Mδ) genes.

To determine the phylogenetic relationship of differentially expressed MADS-box genes in sweet cherry floral buds and those MADS-box genes found in their genome, we constructed phylogenetic tress with the full length protein sequences of Type I and Type II MADS-box genes from sweet cherry, Arabidopsis and *Prunus mume*.

The *P*.*avium* Type I MADS-box genes include members from the Mα, Mβ, Mγ and Mδ subclades (**S5 Fig**). Fourteen, two, sixteen and five genes from *P*. *avium* belonged to the Mα, Mβ, Mγ and Mδ subclades, respectively. Ten subfamilies of Type I genes were present in the *P*. *avium* genome (except the AGL39/AGL74 and AGL103 subfamilies). The two Type I MADS-box transcription factors differentially expressed during S1-S4 bud development belonged to the AGL67 subfamily from the Mδ subclade. Based on the phylogenetic dendrogram, Type I MADS-box genes from *P*. *avium* were grouped with their putative orthologous genes from *P*. *mume* first, and then grouped with their homologs from Arabidopsis.

**Fig 3:**
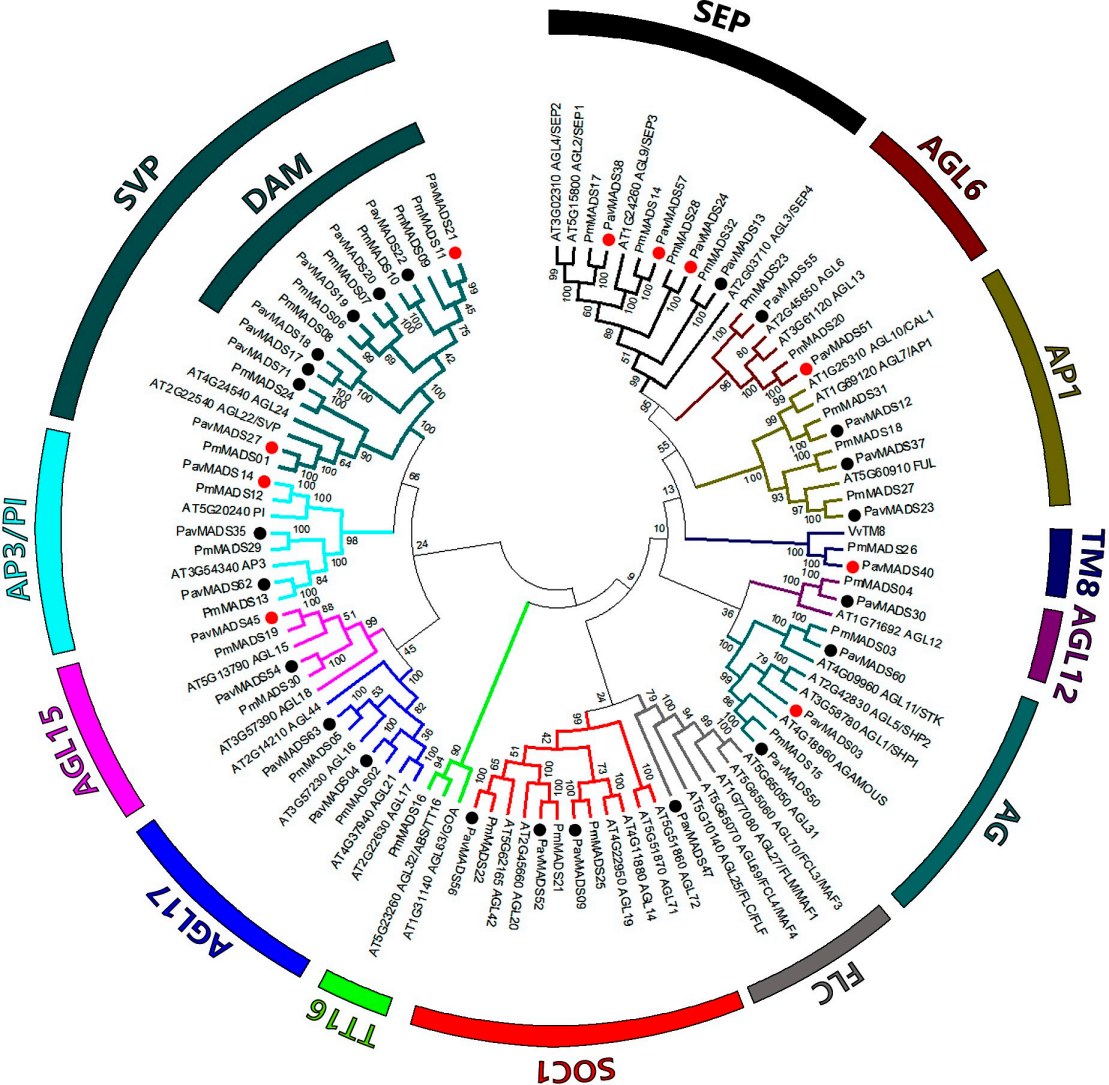
Phylogenetic relationships among sweet cherry Type II MADS-box proteins. The MADS-box subfamilies and the DAM-like proteins are indicated. The phylogram was generated with the MEGA 6.0 program from the multiple alignments of the deduced amino acid sequences from *P*. *avium* (Pav), *Arabidopsis* (At) and *P*. *mume* (Pm) MADS-box proteins. Bootstrap values from 1000 replicates were used to assess the robustness of the tree. Black and red dots indicate *P*. *avium* proteins. Also, red dots indicate *P*. *avium* genes analyzed by qPCR.

The *P*. *avium* Type II MADS-box genes (MIKCc clade), contain members from 12 established subfamilies[35] (**Fig 3**). Five out the 12 Type II subfamilies contained similar numbers of genes in Arabidopsis, *P*. *mume* and sweet cherry. Important exceptions occurred in the two subfamilies that play a pivotal role in Arabidopsis vernalization and flowering time: SVP/AGL24 and FLC. In Arabidopsis, the SVP/AGL24 subfamily contains only the two eponymous genes. In sweet cherry, the subfamily is expanded to eight genes. The phylogenetic analysis revealed that the *P*. *avium* SVP subfamily could be subdivided in two clusters, one composed by six putative DAM-like genes and the other, by two genes orthologs to Arabidopsis SVP. To deep insight about how the SVP/DAM subfamily is organized in sweet cherry genome, we constructed a phylogenetic tree with the eight SVP/DAM-like genes from *P*. *avium* and SVP/DAM-like genes from *P*. *mume*[30], *P*. *persica*[36] and *Rosaceae* species (pear and apple) (**S6 Fig**). Two and six sweet cherry genes were clustered with SVP-like and DAM-like genes from other stone fruit tree species, respectively. Orthologs of *Prunus* DAM1 (*PavMADS21*), DAM2 (*PavMADS22*), DAM4 (*PavMADS17* and *PavMADS18*), DAM5 (*PavMADS20*) and DAM6 (*PavMADS19*) are present in the sweet cherry genome but no one ortholog of DAM3 was found. Furthermore, DAM genes from stone fruit trees were clustered together and separated from DAM genes from *Pyrus sp*. and *Malus sp*. suggesting divergence between the *Rosaceae* DAM protein sequences.

Conversely, the FLC subfamily contains six members in Arabidopsis (FLC and MAF1-5) but only a single member in sweet cherry (*PavMADS47*). Furthermore, sweet cherry genome contains a member from TM8 subfamily (*PavMADS40*) which is absent in Arabidopsis genome. Also, there were three *P*. *avium* homologs each for the *Arabidopsis* APETALA1/FRUITFULL (AP1/FUL), AGAMOUS and the SUPPRESSOR OF OVEREXPRESSION OF CONSTANS 1 (SOC1) subfamilies, four for the SEPALLATA (SEP) subfamily and two each for the AGL6 and AGL17 subfamilies.

The Type II MADS-box transcription factors differentially expressed during S1-S4 bud development belonged to 10 out the 12 subfamilies. The most represented Type II MADS-box subfamily was the SVP (*PavMADS17*, *PavMADS19*, *PavMADS20*, *PavMADS21* and *PavMADS27*) with five DEGs whereas three DEGs from SEP (*PavMADS13*, *PavMADS24* and *PavMADS38*) and PI (*PavMADS14*, *PavMADS35 and PavMADS62)* subfamilies were found. The AGL6, TM8, AG, FLC, AGL12, AGL15 and APETALA 1 subfamilies were represented each by one DEG.

### Expression analysis of *MADS*-box genes

As a first approach to ascertain the role of DEGs MADS-box transcription factor genes in sweet cherry development, their temporal and spatial expression patterns were analyzed by qRT-PCR. Total RNA samples were isolated from vegetative and reproductive organs in different, but specific, stages of development viz. flowering induction and flower whorls development represented by S1-S4 floral buds, S1-adjacent leaves, organs of flower at anthesis (sepals, carpels, petals and stamens), inmature fruits and mature fruits. We selected twelve candidate genes for qPCR study that represent two types and 9 subfamilies of MADS-box gene family.

**Fig 4:**
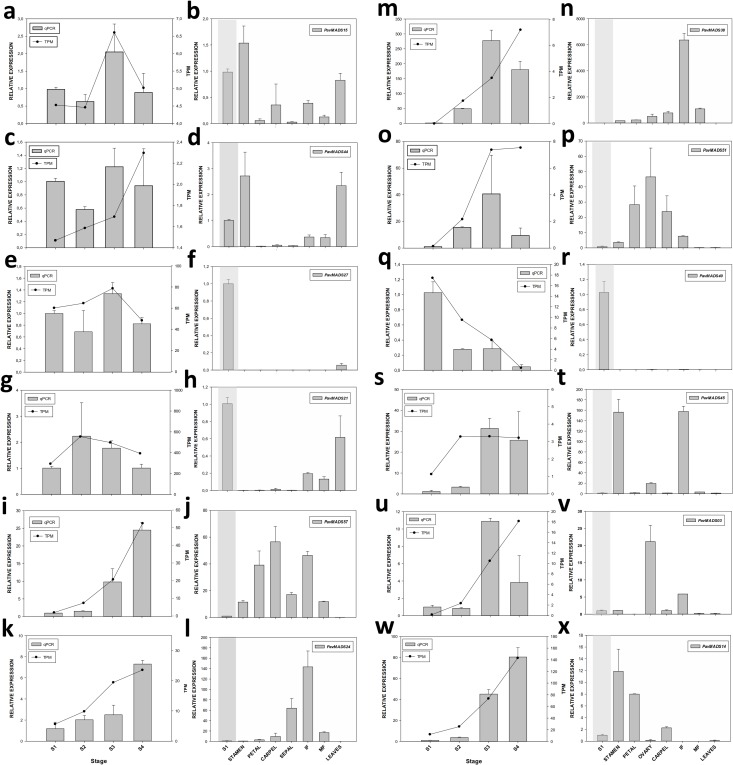
Expression analysis of sweet cherry MADS-box genes in floral buds and various organs by qRT-PCR. **a-b** *PavMADS15;* **c-d** *PavMADS44;* **e-f** *PavMADS27;* **g-h** *PavMADS21;* **i-j** *PavMADS57;* **k-l** *PavMADS24;* **m-n** *PavMADS38;* **o-p** *PavMADS51;* **q-r** *PavMADS40;* **s-t** *PavMADS45;* **u-v** *PavMADS03;* **t-x** *PavMADS14*. Floral buds during flowering induction (S1) and development of floral whorls (S2-S4) were analyzed. Each collection point was performed between the 14th and the 16^th^ day of every month. Expression pattern of MADS-box genes in vegetative organs (leaves) and reproductive organs (Stamen, Petal, Sepal, Carpel, IF inmature fruit and MF mature fruit at harvest time) were also analyzed. The reproductive growth stages analyzed are in agreement with the extended BBCH’s scale(1) and are the following: Flower whorls were collected from flowers at anthesis stage 65, IF stage 77 and MF stage 89. Gray bars represent the relative expression values obtained by qPCR and the black solid line represents the expression level of genes according to the TPM value. The expression levels of the analyzed genes were normalized against that of *TEF2*. Data are mean ± SD (n = 3).

Two Type 1 MADS-box genes, *PavMADS15* (**Fig 4A and 4B**) and *PavMADS44* (**Fig 4C and 4D**), that belong to the Delta subclade were differentially expressed during S1-S4 bud development. In floral buds, both genes showed a similar expression pattern with a maximum in their expression in S3 buds. Also, *PavMADS15* and *PavMADS44* were higher expressed in stamens in flowers at anthesis and during fruit development. Furthermore, both genes were highly expressed in leaves suggesting also a role during the development of vegetative organs.

The DEGs that belong to the SVP subfamily, *PavMADS27* was phyllogeneticaly related to SVP whereas *PavMADS21* and *PavMADS22* seems to be orthologs of *DAM1* and *DAM2*, respectively. The *SVP*-like gene, *PavMADS27*, increased their expression in S3 during the floral primordia development (S1-S4) and it also was expressed in leaves (**Fig 4E and 4F**). The *PavMADS21* (*DAM1*-like) transcripts increased until S2 and a reduction of their transcript levels was evident as the floral bud development stages progressed. Also, *PavMADS21* was expressed in fruits but mainly in leaves (**Fig 4G and 4H**).

Three E-class *SEP*-like genes differentially expressed in the transcriptomes of floral buds were homologs of Arabidopsis *SEP1/2* (*PavMADS38* and *PavMADS24*) and *SEP3* (*PavMADS57*). During the floral bud development the three *SEP*-like genes showed a similar expression pattern with an augment in their transcripts from S1 to S4 with the concomitant development of flower primordia (S2-S4)(**Fig 4I, 4K and 4M**). As a rule, the *SEP*-like genes were higher expressed in flowers (sepals, stamens, petals and carpels) and fruits than in S1 buds. In fruits, *SEP*-like genes were higher expressed in immature than in mature fruits. The SEP-like transcripts were undetected in leaves (**Fig 4J, 4L and 4N**). Another putative E-class gene and member of AGL6 subfamily was *PavMADS51*. This gene was clustered with two members of the Arabidopsis *eu*-AGL6 clade, *AGL6* and *AGL13*, but separated from AGL6-like genes, *PavMADS55* and *PmMADS23*. *PavMADS51* transcripts showed a 40-fold increase from S1 to S3 stages (**Fig 4O**), concomitant with the development of floral whorl primordias, and then their expression in S4 decreased. Furthermore, *PavMADS51* was expressed in all flower organs but mainly in carpels. During fruit development, *PavMADS51* was expressed mostly in immature fruits. *PavMADS51* expression was barely detected in vegetative organs (leaves) (**Fig 4P**).

The TM8-like gene, *PavMADS40*, was almost exclusively expressed in floral buds with their maximum of expression during flowering induction (S1) and a 7-fold reduction of their transcript levels in S2-S4 floral buds (**Fig 4Q**). Transcripts of *PavMADS40* were undetected in vegetative (leaves) or reproductive organs (flowers and fruits) (**Fig 4R**).

The *PavMADS45*, one out the two MADS-box genes that integrated the AGL15 subfamily in *P*. *avium*, was differentially expressed in S1-S4 buds. The *PavMADS45* transcripts showed a maximum in the S3 buds showing a 30-fold increase compared to S1 (**Fig 4S**). The sharply increase in *PavMADS45* expression was associated with the development of flower whorl primordias. In other reproductive organs, such as flowers, the sweet cherry *AGL15*-like gene showed higher expression levels in stamens (>140-fold increase compared to S1 buds) but also was expressed in the fourth whorl (ovary). Noteworthy, *PavMADS45* showed a >140-fold higher expression in green fruits than in S1 buds or mature fruits (**Fig 4T**). In leaves, *PavMADS45* was barely detected.

Three putative C-class MADS-box genes from the *AGAMOUS* subfamily were found in the sweet cherry genome (*PavMADS03*, *PavMADS50* and *PavMADS60*). *PavMADS03* gene was differentially expressed in S1-S4 buds and it was phyllogeneticaly clustered with Arabidopsis *AGL1/SHATTERPROOF 1* (*SHP1*) and Arabidopsis *AGL5/SHATTERPROOF 2* (*SHP2*). During floral bud development, the *PavMADS03* transcripts showed a maximum in S3 showing a >10-fold increase between S2-S3 transition and a decrease in their expression in S4 buds (**Fig 4U**). The *SHP*-like gene was mainly expressed in carpels of flowers at anthesis but also a noticeable expression of it in green fruits and anthers was observed (**Fig 4V)**.

The *PavMADS14*, a putative B-class MADS-box gene from the APETALA3/PISTILATA (AP3/PI) subfamily, was DEG in floral buds. *PavMADS14* expression showed a sharply increase in S3 and S4 buds with concomitant development of flower whorls primordial (**Fig 4W)**. In flowers at anthesis, *PavMADS14* was highly expressed in stamens, petals and sepals and a higher amount of their transcripts in the mentioned organs than in S1 buds was found. The expression of this *AP3/PI*-like gene was barely detected in fruits and leaves (**Fig 4X)**.

### Validation of RNA-seq by qRT-PCR

To validate RNA-seq, we performed cDNA synthesis using the total RNAs for RNA-seq and used the cDNA as a template for qRT-PCR. The expression levels of 12 selected transcripts were detected in S1-S4 floral buds (**Fig 4)**. Selected transcripts codifying for *MADS*-box like genes were found through RNA-Seq analysis to have different expression patterns. The 12 selected transcripts had similar expression patterns between RNA-Seq and qRT-PCR, suggesting reliable expression data by RNA-Seq.

### Gene Regulatory Network (GRN) during floral transition

**Fig 5:**
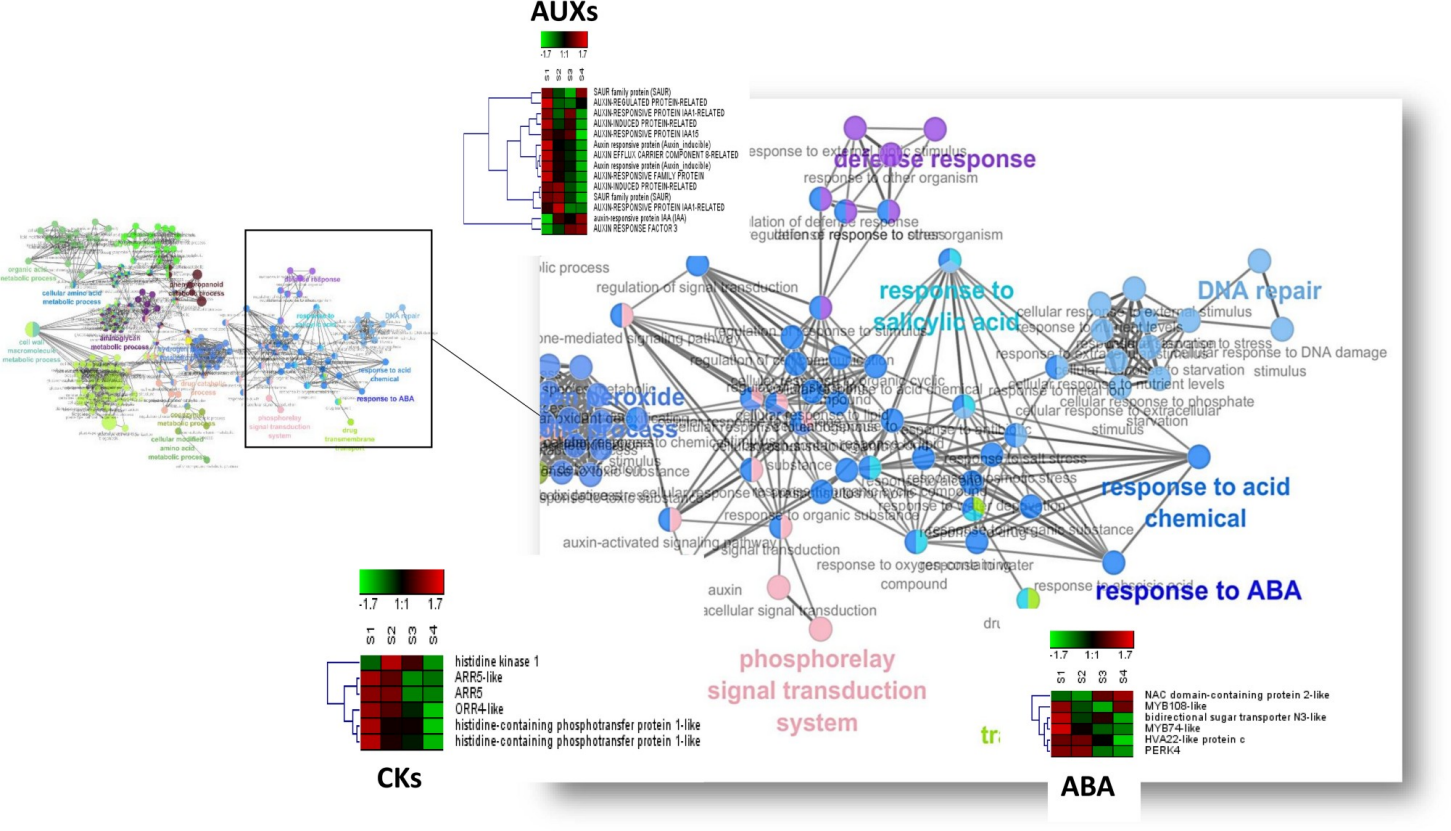
GRN and enrichment analysis of biological processes of DEG during flower bud development (S1-S4). Zoomed-in of larger cluster to show a close-view of hormone nodes. The expression patterns (heatmap) of candidate genes that compose the nodes is also showed. ABA: abscisic acid-Response to ABA; CKs: Cytokinin-Phosphorelay transduction system; AUX: auxin-response to acid chemicals. The GRN was visualized by Cytoscape 3.7.1 and the GO enrichment analysis performed with ClueGO[37].

In order to reveal transcriptome signatures of the floral bud development in sweet cherry we built a co-expression gene network considering the DEGs from the S1-S4 bud development stages (**Fig 5**). Then, we did a Gene Ontology (ClueGO)[37] enrichment analysis to know if the gene-clusters found are functionally related with biological processes potentialy involved in the floral bud differentiation in this species[38]. Our analysis revealed that the larger cluster was enriched in several biological processes, being the most representatives “response to abscisic acid”, “phosphorelay transduction system”, “response to acid chemicals (including a node of respone to auxin)”, “response to salycilic acid”, cell wall associated processes (“cell wall macromolecule metabolic process” and “cell wall biogénesis”), regulation of metabolic processes (“cellular amino acid” and “organic acid”) and “protein glycosylation” It is outstanding to mention the enrichment in genes with roles in the signal transduction of hormones such as abscisic acid, cytokinin and auxins suggesting that these hormones and their related genes could play an important role during the floral bud differentiation in sweet cherry (**Fig 5**). Within the auxin-related genes, the repressor *IAA1* and *IAA15*-like genes disminished their expression from S1 to S4 and this was related with an increase in the expression of *ARF3*-like gene transcription factor. An *AUXIN-EFFLUX CARRIER 8*-like gene was mainly expressed in S1 during flowering induction. ABA-related genes such as bidirectional sugar transporter *SWEET15*-like, Proline-rich receptor-like protein kinase *PERK4*-like, *MYB74*-like gene transcription factor and *HVA22*-like were higher expressed in S1 or S2 buds and a decline in their expression as the floral bud development progressed. The cytokinin-related genes (*HISTIDINE KINASE 1*, *AAR15*, *ARR16*, *AHP1*-like and *AHP4*-like) and were higher expressed during the S1-S3 than in S4 buds suggesting a role of this hormone during the first stages of floral bud differentiation (**Fig 5**). The enrichment analysis suggests that the highly connected genes from the largest cluster in the network are involved in the regulation of complementary processes triggered by floral transition. We also perform enrichment analysis on the other clusters from the network. The second larger cluster was enriched in “peptide metabolic process”, “translation” and “phosphorous metabolic processes”. A third cluster was enriched in “ion transport” (including several ABC transporter-like genes) and “organic subtances transport” (including several bidirectional sugar transporter *SWEET*-like genes). A fourth cluster was enriched in development process involved in reproduction (including *FLOWERING LOCUS T*, *BROTHER of FT and TFL1* and, *MOTHER of FT and TFL1*-like genes). Other four smaller clusters were enriched in “generation of precursor metabolites and energy”, “cellular amino acid metabolic process”, “organelle organization” and “response to temperature stimulus”, respectively.

### Hormonal profile of buds and bud-adjacent leaves during the floral transition (S1-S4 stages)

**Fig 6:**
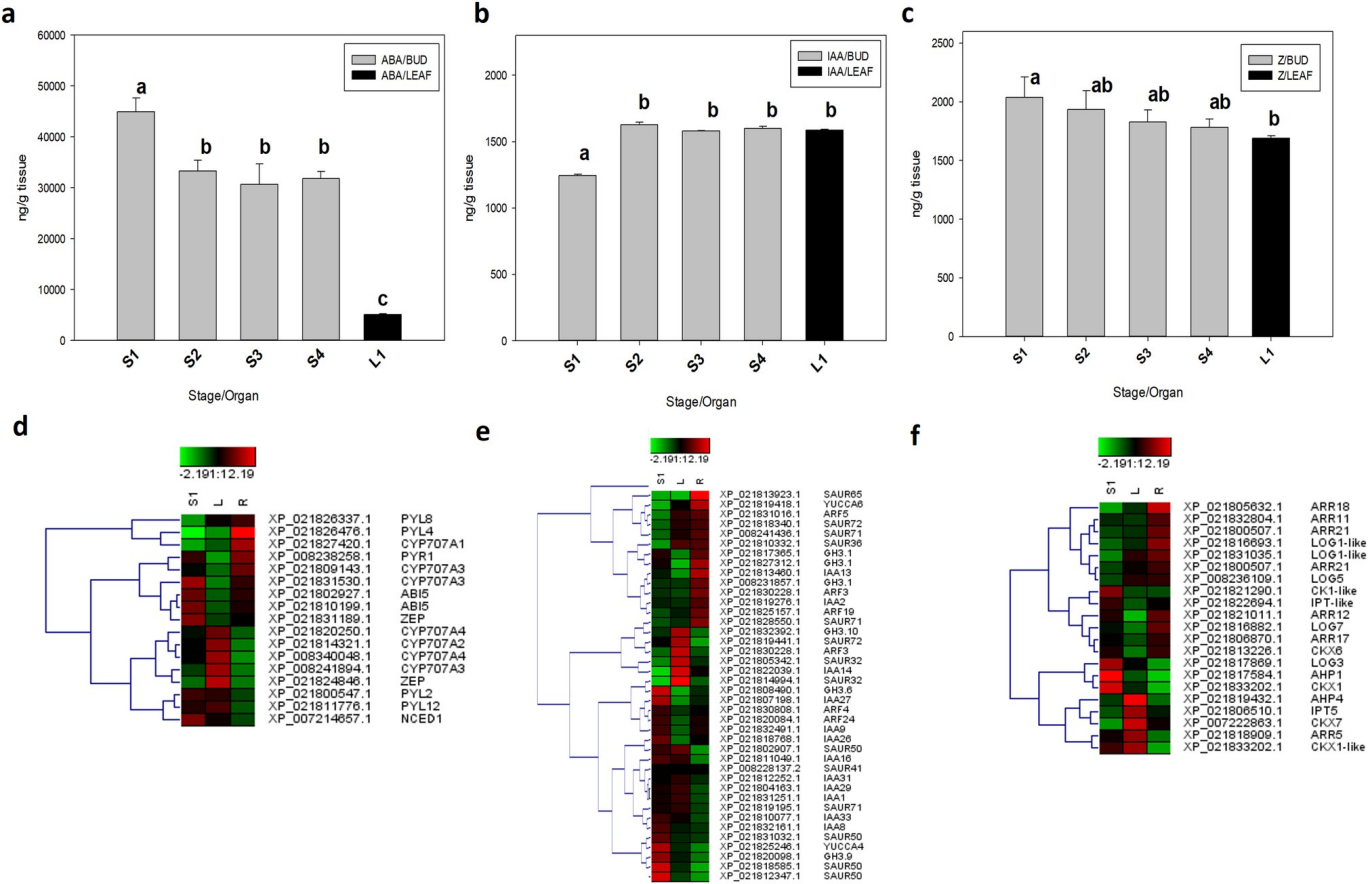
Dynamic changes in abscisic acid (ABA), indol acetic acid (IAA), and zeatin (Z) content and expression of key genes related to hormone metabolism and signalling pathways during *P*. *avium* cv. Bing floral bud differentiation and S1-adjacent leaves. **a-d:** ABA; **b-e:** auxin (IAA); **c-f:** cytokinin (Z). The gray bars represent the hormone content in S1-S4 floral buds and the black bar represents the hormone content in S1-adjacent leaves (L1). Data are mean ± SD (n = 3). The heat maps represent the log2 fold changes (FDR≤0.05) of DEGs related to hormone metabolisms. Red and Green colors represent up- and down-regulated genes, respectively. Two biological replicates for each developmental stage (S1: bud) or organ (L1: leaves; R: roots) is showed. Scale, representing the signal values, is shown at the top of the Fig.

We found hormonal-related signatures associated to ABA, auxin and citokinin signal transduction in the floral bud transcriptomes. Thus, we analyzed the endogenous contents of ABA, auxin (*Indol Acetic Acic*, IAA) and cytokinin (*zeatin*, Z) in buds at S1-S4 stages and in S1 bud-adjacent leaves (vegetative organ) by HPLC-ESI-MS/MS (**Fig 6**). Interestingly, ABA content was high in the S1 buds, at flowering induction, and then ABA content decreased by ~26% in S2-S4 buds during the flower whorls development. Noticeably, ABA content in S1 buds was higher than in their adjacent leaves (vegetative organ) (**Fig 6A**).

IAA content was low in S1 buds, at flowering induction, and then showed an increase of ~30% in S2-S4 buds during flower bud development. In S1 bud-adjcent leaves the IAA content was higher than in S1 buds (**Fig 6B**).

Z content showed a maximum in S1 buds and then it gradually decreased during the floral whorls development. Z content was higher in S1 buds than in their adjacent leaves (**Fig 6C**).

Since we found significant differences in the ABA, IAA and Z contents in S1 buds and S1 bud-adjacent leaves, we scutrinized the DEGs in these organs and roots in order to find genes related with their synthesis, perception, degradation and/or signal transduction. The important genes for ABA biosynthesis, *ZEP-like* (XP_021831189.1) and *NCED1* genes were higher expressed in S1 than in leaves, although transcripts of an other *ZEP*-like gene (XP_021824846.1) were preferentially accumulated in leaves (**Fig 6D**). In the ABA signal transduction pathways, *PYL2*, *PYL12*, *PYR1*, as ABA receptors were higher expressed in S1 buds than in leaves. Also, genes codifyng the ABI5 transcription factor were preferentially expressed in S1 buds. ABA 8'-hydroxylase is a subfamily of P450 monooxygenases and is encoded by CYP707A genes. CYP707A catalyzes the committed step in the major ABA catabolic pathway. We found three genes (CYP707A2, CYP707A3 and CYP707A4) preferentially expresed in leaves whereas one gene (CYP707A3) expressed in S1 buds suggesting that the ABA catabolic machinary may be more active in leaves.

In the auxin metabolism (**Fig 6E**), the *YUCCA* and *TAR2* (*TRYPTOPHAN AMINOTRANSFERASE RELATED*) genes positively regulate the IAA biosynthesis. A *YUCCA4* and two *TAR*-like genes were mainly expressed in S1 buds. Auxin-efflux carriers *PIN3-*like (XP_021815515.1) and *PIN5*-like (XP_021804584.1) were preferentially expressed in S1 buds whereas another *PIN3*-like genes (XP_021815553.1) was higher expressed in leaves than in S1 buds. Additionally, three members of the *GH3* family auxin-responsive genes, *GH3*.*1*, *GH3*.*6* and *GH3*.9, which codified for IAA-amido synthase were mainly expressed in S1 buds. In the auxin-activated signaling pathway, *SMALL AUXIN UP RNAs* (*SAURs*) are the largest family of early auxin response genes. The *SAUR50* and *SAUR71* genes were mainly expressed in S1 buds whereas *SAUR41* and *SAUR72* were concomitantly expressed in buds and leaves. Outstanding, several *Auxin/INDOLE-3-ACETIC ACID* (*Aux/IAA*) transcriptional repressors (*IAA1*, *8*, *9*, *18*, *26*, *29*, *31* and *33*) were up-regulated in S1 buds whereas only *IAA14* was differentially expressed in leaves. The *AUXIN RESPONSE FACTOR* (*ARF*) transcription factors *ARF4* and *24* were highly expressed in S1 buds whereas another *ARF3*-like was up-regulated only in leaves.

The *isopentenyltransferases* (*IPT*) and the “Lonely guy” (*LOG*) genes, which are associated with the cytokinin biosynthesis, were differentially expressed in S1 buds and leaves (**Fig 6F**). The *IPT*-like (XP_021822694.1) and *LOG3* were mainly expressed in buds whereas *IPT5*, *LOG1* and *LOG5*, were preferentially expressed in leaves. The signal transduction histidine kinases, *CKI1* and *AHP1*, were mainly expressed in buds but *AHP4* transcripts were more abundant in leaves. The *response regulators* were expressed mainly in leaves (*ARR18* and *ARR21*) or buds (*ARR12*) or in both organs (*ARR5*). The cytokinin oxidase (*CKX*) genes, *CKX1* and *CKX6* higher expressed in buds and the transcripts of another *CKX1*-like were equally accumulated in S1 buds and leaves.

## Discussion

### Sweet cherry floral buds have a distinctive transcriptome during flowering induction and flower organogenesis

The flowering induction and flower bud formation on sweet cherry trees play an important role during their life cycle. Beside their biological importance, the flowering induction and many factors related to floral biology of sweet cherry trees influence productivity and they determine to a considerable extent the success of commercial orchards[4,5]. Recently, expression profiles have been performed in buds from several members of the *Prunus* genus. However, most of them have been focused in dormancy transitions[39,40]. In this study, we reported a comprenhensive transcriptome study of four development stages of floral bud differentiation during flowering induction and the development of flower whorls in *P*. *avium* cv. Bing floral buds. As a step toward functional annotation of genes required for floral initiation and flower development we compared these information with transcriptomes of buds at dormancy and vegetative tissues, including leaves and roots.

The comparisons of floral bud transcriptomes during flowering induction (S1), flower whorls development (S2-S4) and dormancy (D) revealed that many DEGs exhibited developmental stage-specific expression. Furthermore, PCA analysis showed the distinctness of S1-S4 from D bud transcriptomes, suggesting the presence of different transcriptional programmes acting in floral buds during the first growing season and dormancy. Furthermore, when the transcriptome of floral buds (S1) was compared with the transcriptomes of vegetative tissues (leaves) around 5% of DEGs were exclusively up-regulated in S1 buds, perhaps as consequence of leaf-like bracts on the floral buds. This is in agreement with the reported in other woody tree species since floral buds are complex organs (e.g. *Eucalyptus grandis*[29]). Three transcription factors, with homology to *AtMYB16*, *AtWER* and *SPECHLESS* from Arabidopsis were specifically up-regulated in early floral buds (S1). In Arabidopsis, the expression of *AtMYB16* and *SPEECHLESS*[41] is induced in the shoot apex during floral transition associated with assymetric cells division whereas *WER*[42] play a role in the epidermal cell fate determination. Other genes that were exclusivelly expressed in S1 buds are related to plant cell wall metabolism (*XYLOGLUCAN ENDOTRANSGLUCOSYDASE* and *PECTINESTERASE*), translation (*60S RIBOSOMAL PROTEIN L15*), biotic (*RPP13*) and abiotic (*HEAT SHOCK PROTEIN 90–3*) stress reponses and they may play important roles in the differentiation process of this actively growing buds.

### Major transcription factors in floral bud transcriptomes

Transcription factors play important roles in the regulation of downstream targets, making them crucial to diverse biological processes in plant growth, development, and stress responses[43,44]. The enrichment analysis of DEG-based GOs during the flowering transitions in *P*.*avium* revealed the GO “nucleic acid binding transcription factor activity” suggesting an important role of transcription factors in the floral bud differentiation process. Around six per cent (6%) of the 2,982 DEGs found during floral transition (S1-S4 and D) were predicted transcription factors. These genes belonged to diverse gene families (e.g. *bHLH*, *NAC*, *MYB* and *MADS-box*), many without reported role in floral development of stone fruit trees. In general, most of *bHLH*, *NAC* and *MYB* genes were preferentially expressed during flowering induction (S1) and a decline in their amount of transcripts during the development of floral whorls (S2-S4) was observed suggesting novel roles for these transcription factor in flowering induction and flower organogenesis in this species. In Arabidopsis, the *bHLH35*, *49*, and 94 are highly expressed in the shoot apex during the floral transition which agree with the expression pattern of *PavbHLH35*, *PavbHLH49 and PavbHLH94* in *P*. *avium* floral buds and may have similar functions during flowering. The *MYB* genes play roles in petal and stamen development in *A*. *thaliana* (*AtMYB6*, *106* and *111*[45]) and their homologs in *P*. *avium* are mainly expressed in floral buds (S3-S4) during the develop of mentioned organs suggesting similar roles. Furthermore, the TFs could also play important roles transducing and/or integrating signals from the environment (e.g. *AtMYB4* regulates the accumulation of UV protective napoylmate and their homolog *PavMYB4*-like was expressed in S1-S3 buds during the summer when UV radiation is high[46]) and hormones (e.g. *AtMYB41* mediates ABA signalling and *PavMYB41*-like gene was mainly expressed in S1 buds when ABA content is high[45]) controlling thus the sweet cherry floral bud transitions.

### The MADS-box gene family plays an important role during the floral bud development

The *MADS*-box gene family stand out among the transcription factors differentially expressed in sweet cherry floral buds suggesting a key role in the flowering transitions in this species. In plants, *MADS*-box genes are master regulators of developmental processes. Beyond their critical role in flower development, *MADS*-box genes are important for flowering time control, inflorescence architecture, pollen development, seed/fruit development, and root development[47]. We identified 78 MADS-box proteins in the *P*. *avium* genome which were divided into Type I (Mα, Mβ, Mγand Mδ) and Type II (MIKC^c^) based on the *MADS*-box proteins from Arabidopsis. Furthermore, *P*. *avium* MIKC^c^ proteins were further subdivided in twelve subfamilies. The size of the *P*. *avium MADS-box* gene family is smaller than Arabidopsis (106 genes) but similar to peach [36] and Japanese apricot (*P*. *mume*)[30]. Expression analysis by qPCR of two Type 1-Delta subclade *MADS*-box genes (*PavMADS15* and *PavMADS44*) showed that they are mainly expressed in S3 buds and stamens of flowers at anthesis but also in leaves. In Arabidopsis, *AtAGL30* and *AtAGL65*, the homologs of the *PavMADS15* and *PavMADS44* genes, respectively, are highly expressed in pollen and they act as heterodimer complexes (with AGL66 or AGL104) that seem to be major regulators of pollen maturation programs[48]. In *P*. *mume*, *PmMADS47* and *PmMADS68*, homologs of *AtAGL30* and *AtAGL65*, respectively, are expressed mainly in flowers but also in fruits and vegetative organs (leaves, stem and roots)[30]. During *P*. *avium* floral bud development, *PavMADS15* and *PavMADS44* were mainly expressed in S3 buds when the stamen primordias started their differentiation and this agree with the observed in *Eucalyptus*[29] suggesting that these genes play a role in the early development of androecium in trees.

We found eight genes in the SVP subfamily, two genes belong to the SVP-clade and six genes to the DAM-clade. The sweet cherry DAM proteins were phyllogeneticaly clustered with orthologs from other *Prunus* species but separated from other *Rosaceae* DAM proteins from *Malus* and *Pyrus* suggesting an evolutionary diversification. Recently, Falavigna et al. (2019)[49] found similar results in the phylogenetic analysis of SVP and DAM proteins from temperate fruit trees suggesting a neofunctionalization between *DAM* and *SVP*-like genes and, a subfunctionalization within the *DAM-like* genes. In the *P*. *avium* floral bud transcriptomes, we found four DEGs that belong to the SVP subfamily and we analyzed in detail the expression of *PavMADS21* and *PavMADS27*, homologs of *DAM1* and *SVP*, respectively. Both genes were preferentially expressed in S2-S3 floral buds during the floral whorls development. In Arabidopsis, SVP regulates the pattern of floral organ development together with other MADS-proteins (AGL24 and AP1)[50]. The ectopic expression of *SVP*-like genes from woody angiosperms (e.g. kiwi and Japanese apricot) in heterologous systems alters flowering time and the pattern of floral organogenesis[51,52] suggesting a molecular function similar to the one of Arabidopsis *SVP* [49]. Our results suggest a role for *PavMADS21* and *PavMADS27* during floral organogenesis. Because sweet cherry buds are complex organs, *in situ* analysis of *PavDAMs* and *PavSVPs* transcripts and the unraveling of interaction map of these proteins will provide a more accurately evidence of *DAM*-like and *SVP*-like genes spatial expression and function.

A single copy gene seems to conform the TM8 subfamily in *P*. *avium* and this agree with the observed in peach and Japanese apricot genomes[30,36]. The sweet cherry TM8-like gene was exclusively expressed in flower buds (S1) during the flowering induction period but not detected in anyother organ analyzed. In *P*. *persica* (*PpeMDAS35*) and *P*. *mume* (*PmMADS26*) the TM8-like genes were expressed in flowers, pistil and fruits suggesting a role in the development of such organs[30,36]. The TM8 subfamily is absent from the Arabidopsis genome and none of the genes in this subfamily have been functionally characterised in any *Prunus* species[53]. The expression patterns of TM8-like genes in *Prunus* suggest the neofunctionalization of these genes in stone fruit trees.

Two genes constitute the *AGL15* subfamily in *P*. *avium*. A similar number of genes have been observed in Arabidopsis (*AtAGL15* and *AtAGL18*) and *Prunus* species[30,36]. The sweet cherry *AGL15*-like gene, *PavMADS45*, was expressed in floral buds during the flower whorls differentiation but mainly in carpels, stamens and inmature fruits. In peach, the *PavMADS45* orthologs (*PpeMADS17* and *PpeMADS30*) were mainly expressed in pollen whereas in Japanese apricot (*PmMADS19* and *PavMADS30*) were expressed in flowers, fruits and leaves[30,36]. In Arabidopsis, *AGL15* and *AGL18* act redundantly as floral repressor contributing to the control of the transition from the vegetative to reproductive phase through the regulation of *FLOWERING LOCUS T* [48]. The *AGL15* subfamily genes, along with *SVP* and *AGL24*, are necessary to block premature activation of *SEPALLATA 3* and the expression of reproductive programs during the vegetative phase[54]. *PavMADS45* and *SVP*-like gene expression increased until S3 and their down-regulation in S4 buds was concomitant with a sharply increase in the *SEP3*-like gene expression suggesting that the *SVP-AGL15* repressive module could also be acting during floral bud organogenesis in sweet cherry.

Based on the ABCDE genetic model of floral organ identity[55], we analyzed by qPCR the expression of class B (*PavMADS14*, AP3/PI subfamily), C (*PavMADS03*, *SHP1*) and E-like genes (*PavMADS24*, *PavMADS38* and *PavMADS57*, *SEPs* and PavMADS51, *AGL6*) of MADS-box family and as a rule they were mainly expressed in S3-S4 floral buds suggesting an important role during the floral organogenesis in *P*. *avium* cv. Bing. As their *A*. *thaliana* homologs, expression of BCE-like genes was mainly restricted to floral buds, flower organs and inmature fruits and they transcripts were not detected in leaves. *In situ* analysis of floral homeotic “ABCDE” MADS-box transcripts will provide a more accurately evidence of their spatial expression and function during floral bud organogenesis.

### ABA, cytokinin and IAA pathways are active during flowering induction and flower organogenesis in sweet cherry trees

Plant hormones stand out among the endogenous signals controlling the flowering process in plants. Plant hormones interfere with flowering induction and other plastic processes such vegetative growth, dominance phenomena, fruit set and growth, stress situations, among others[56]. However, the molecular basis of their actions during the flowering induction and floral bud organogenesis in *P*. *avium* cv. Bing has been little explored. Our analysis of gene regulatory networks and hormone contents in four stages of differentiation of floral buds revealed a potential role for ABA, cytokinin and auxins during the flowering induction and floral bud organogenesis in *P*. *avium* cv. Bing. During flowering induction, the IAA content was lower in buds (S1) than leaves and then it increased in buds during the organogenesis (S2-S4). The lower concentration of auxin in S1 buds was associated with a dome-like meristem without an evident differentiation of flower primordium. In Arabidopsis, the AUXIN RESPONSE FACTOR5/MONOPTEROS (ARF5/MP) has a central role translating local auxin concentration into specific gene expression outputs and flower initiation. In the absence of auxin, MP activity is inhibited by the physical interaction between MP and Aux/IAA proteins, this represses transcription of downstream target genes involved in flower formation[15]. Auxin sensing promotes the degradation of Aux/IAA12, resulting in MP-dependent transcriptional activation of target genes such as *LEAFY* (*LFY*), which specifies floral fate, and to two transcription factors, *AINTEGUMENTA* (*ANT*) and *AINTEGUMENTA-LIKE6/PLETHORA3* (*AIL6/PLT3*), key regulators of floral meristem outgrowth[15] [53]. These results suggest that upregulation of *LFY*, *ANT*, and *AIL6*/*PLT3* by MP contributes to flower primordium initiation. Our results show that several *Aux/IAA*-like genes were mainly expressed S1 buds and it is tempting to hypothesize that they could play a role in repress the transcriptional program(s) that determine the flower initiation. When auxin concentration increased (S2-S4 buds), the Aux/IAA proteins are degradated and ARF-like protein(s) could promote the expression of their target genes such as *LEAFY* (*LFY*). We observed the co-expression of *LFY*-like and *ANT*-like genes in S2 buds when flower primordias have been developed. Taken together, our results suggest that the auxin-mediated differentiation of flower primordia in sweet cherry may depend on a genetic circuit similar to that described in Arabidopsis. The transcriptional control of *ARF* transcription factors appears to play a key role in specifying and maintaining distinct auxin responses in certain tissues or at specific developmental stages[57,58]. We found several *ARF*-like genes differentially expressed during the floral bud development (*ARF1*, *2*, *3*, *4*, *6*, *8* and *18*) suggesting that these genes may be involved in the process of flowering induction and organogenesis in *P*. *avium*.

The phytohormone cytokinin plays diverse roles in plant development, influencing many agriculturally important processes, including growth, nutrient responses and the response to biotic and abiotic stresses[59]. We found higher cytokinin content in buds during flowering induction (S1 buds) than in S1-adjacent leaves and a decrease in cytokinin content during the flower organogenesis (S2-S4 buds). Also, GRN analysis of DEGs in flower buds showed an enrichment in genes associated with phosphorelay signal transduction system suggesting an active role of cytokinins during flowering induction in sweet cherry. Cytokinins would promote the flowering induction in perennial polycarpic plants such as *P*. *avium*[56]. In sweet cherry (cv. Bing), the exogenous application of 6-BA-benzyladenine (as promalin) increases the number of floral buds in spurs. The proportion of flowering was increased when 6-BA plus paclobutrazol (GAs biosynthesis inhibitor) was applied[60]. In Arabidopsis, cytokinin-mediated flowering under short days required the activation of *TWIN SISTER OF FT* (*TSF*) and the *SOC1* and *FD* functions. Furthermore, Arabidopsis *RESPONSE REGULATOR 5* (*ARR5*) gene together *TSF*, *SOC1* and *FD* are transcriptionally up-regulated when cytokinin is applied exogenously[14]. Among the targets of type-B RRs is the gene encoding the transcription factor *WUSCHEL*, a key regulator of shoot meristem activity, thereby providing a direct link between cytokinin signaling and the regulation of shoot growth and development[59]. We observed an up-regulation of *PavSOC1*, *PavFD* and *ARR5*-like transcription factors in buds during flowering induction and flower primordia development when the higher amount of cytokinin occurs. Also, a cytokinin receptor-like genes (*CK1*-like), homologs of transcription factors *TSF/FT* and *WUSCHEL* were also mainly expressed in S1 buds. Taken together, our results suggest that the mentioned genes and transcription factors, as in Arabidopsis, may play a hub role in the cytokinin-mediated flowering in *P*. *avium* cv. Bing. Recently, Li et al. (2019)[61] showed that *SOC1* and *FD* orthologs are also up-regulted in apple floral buds when the cytokinin 6-BA was applied and suggest that part of an intrinsic genetic network controlling the cytokinin action during the floral transition could be conserved in *Rosaceae*.

The plant hormone abscisic acid (ABA) has multiple functions in regulating plant development and stress responses[62]. The endogenous ABA levels increased in floral buds of sweet cherry trees during the floral induction period and then decreased during floral organogenesis. Furthermore, ABA content was higher in floral buds (S1) than in the S1-adjacent leaves. Transcripts of genes involved in ABA biosynthesis (*ZEP* and *NCED1*), signal transduction (*PYL2*, *PYL12*, *PYR1* and *ABI5*) and catabolism (*CYP707A3*) were found in S1 floral buds and it is tempting to hypothesize that the ABA pathway is active during flowering induction and floral bud organogenesis in *P*. *avium* cv. Bing. The GRN analysis in sweet cherry floral buds also revealed the enrichment of transcripts involved in the ABA-mediated response(s) in plants such as *MYB*-like transcription factors (*MYB74* and *MYB108*, involved in salt stress response in Arabidopsis and regulated by the RNA-directed DNA methylation pathway involving 24-nt siRNAs)[63], receptor kinase *PERK4* (required for the ABA-dependent influx of Ca2+ and normal ABA sensitivity in seeds and roots of *A thaliana*)[64], sugar transporter *SWEET15* (sugar efflux transporters are critical to the movement of sucrose and hexose from source to sink tissues)[65] and *HVA22c*. Outstanding, *AtMYB74*, *AtPERK4*, *AtSWEET15* and *AtHVA22c* genes are regulated by ABA and they are highly expressed in flower buds or in meristem during the floral transition in Arabidopsis. Furthermore, the altered expression of *PERK4* leads to changes in growth and floral organ formation[64]. In others fruit trees, ABA has also been associated with the flowering transition. In Satsuma mandarin (*Citrus unshiu* Marc.), the increase of the ABA levels was consistent with the accumulation of *FT* homolog transcripts as well as with inflorescence development[66]. In apple, flower induction and formation in response to shoot bending seems to be mediated by ABA through photoperiod and circadian pathways[61]. Taken together, our results suggest that ABA could play an important role during flowering induction in *P*. *avium* cv. Bing and polycarpic perennial plants. However, in *A*. *thaliana* ABA is regarded as a general repressor of flowering. The transcription factors ABI4 and ABI5 are involved in ABA signaling and negatively regulate flowering by directly promoting transcription of the flowering repressor, FLC[67]. Exceptionally, **Riboni et al. (2016)**[18] revealed a positive effect of ABA in flowering of *A*. *thaliana*, but it is restricted to extreme environments, known as the drought-escape response, in which plants accelerate flowering before dying[66]. Whether genes of the ABA pathway have acquired novel function(s) related to floral transitions during the evolution of perennial woody plants is still a question that deserves attention.

## Conclusions

This study integrate histological, genetics (RNA-seq) and physiological (hormones) information for the first time in *P*. *avium* cv. Bing to decipher the key signatures associated with flowering induction and flower organogenesis in sweet cheery trees. Our results provide a framework of information about transcription factors, genes and hormones which may help to unravel the complexity of this development proccess and to build a comprehensive model about the floral transition in *P*. *avium* and other woody perennial angiosperms.

## Supporting information

S1 Fig Bud growth and growth rates during floral bud differentiation stage (S1-S4) and dormancy (D) in sweet cherry cv. Bing. a, Length; b, width; c, growth rate of bud length; d, growth rate of bud width.
(DOCX)

S2 Fig PCA analysis of the floral bud transcriptome data of *P*.*avium* cv. Bing. Samples were under five different developmental stages: S1 (flowering induction), S2, S3, S4 (flower whorls development) and D (dormancy).
(DOCX)

S3 Fig Gene ontology term enrichment analysis based on SEA analysis of DEGs from floral buds: The top enriched GO terms are listed. The DEGs from S1-S2, S2-S3 and S3-S4 floral bud transitions were compared. BP: Biological Process; CC: Cellular Component; MF: Molecular Function.
(DOCX)

S4 Fig Expression patterns of selected differentially expressed transcription factors (TF) families in S1-S4 and D floral buds a) hierachical clustering of 24 transcripts codifyng bHLH TFs. b) hierachical clustering of 31 transcripts codifyng MYB TFs. c) hierachical clustering of 31 transcripts codifyng NAC TFs. The heat maps represent the log2 fold changes (FDR≤0.05) of DEGs TFs. Red and Green colors represent up- and down-regulated genes, respectively. Scale, representing the signal values, is shown at the top of the Fig.
(DOCX)

S5 Fig Phylogenetic relationships among sweet cherry Type I MADS-box proteins. The MADS-box subfamilies are indicated. The phylogram was generated with the MEGA 6.0 program from the multiple alignment of the deduced amino acid sequences from *P*. *avium* (Pav), *Arabidopsis* (At) and *P*. *mume* (Pm) MADS-box proteins. Bootstrap values from 1000 replicates were used to assess the robustness of the tree. Black and red dots indicate *P*. *avium* proteins. Also, red dots indicate *P*. *avium* genes analyzed by qPCR.
(DOCX)

S6 Fig Phylogenetic relationships among sweet cherry and *Rosaceae* SVP/DAM proteins. The MADS-box subfamilies are indicated. The phylogram was generated with the MEGA 6.0 program from the multiple alignment of the deduced amino acid sequences from *P*. *avium* (Pav), *Malus x domestica* (Md), *Pyrus pyrifolia* (Ppy), *P*. *mume* (Pm) and *P*. *pseudocerasus* (Pps). MADS-box proteins. Bootstrap values from 1000 replicates were used to assess the robustness of the tree. Black and red dots indicate P. avium proteins. Also, red dots indicate P. avium genes analyzed by qPCR.
(DOCX)

S1 Table Summary of transcriptomic sequencing of *P*. *avium* cv. Bing. (DOCX)

S2 Table A catalog of *MADS*-box genes in sweet cherry. (DOCX)

S3 Table Overview over the quality and quantity of sequencing reads for each sample before and after trimming. (DOCX)

S4 Table List of primers used in qPCR analysis of MADS-box genes. (DOCX)
