# Supplementary material for: Comparative transcriptomic analysis reveals novel roles of transcription factors and hormones during the flowering induction and floral bud differentiation in sweet cherry trees (Prunus avium L. cv. Bing)
Source: PLoS One. 2020 Mar 12;15(3):e0230110. doi: 10.1371/journal.pone.0230110 (PMC7067470; doi:10.1371/journal.pone.0230110)
Supplement: S1 Fig — a, Length; b, width; c, growth rate of bud length; d, growth rate of bud width. (DOCX) [file pone.0230110.s001.docx]

**SUPPORTING INFORMATION**

**COMPARATIVE TRASCRIPTOMIC ANALYSIS REVEALS NOVEL ROLES OF TRANSCRIPTION FACTORS AND HORMONES DURING THE FLOWERING INDUCTION AND FLORAL BUD DIFFERENTIATION IN SWEET CHERRY TREES (*PRUNUS AVIUM* L. CV. BING)
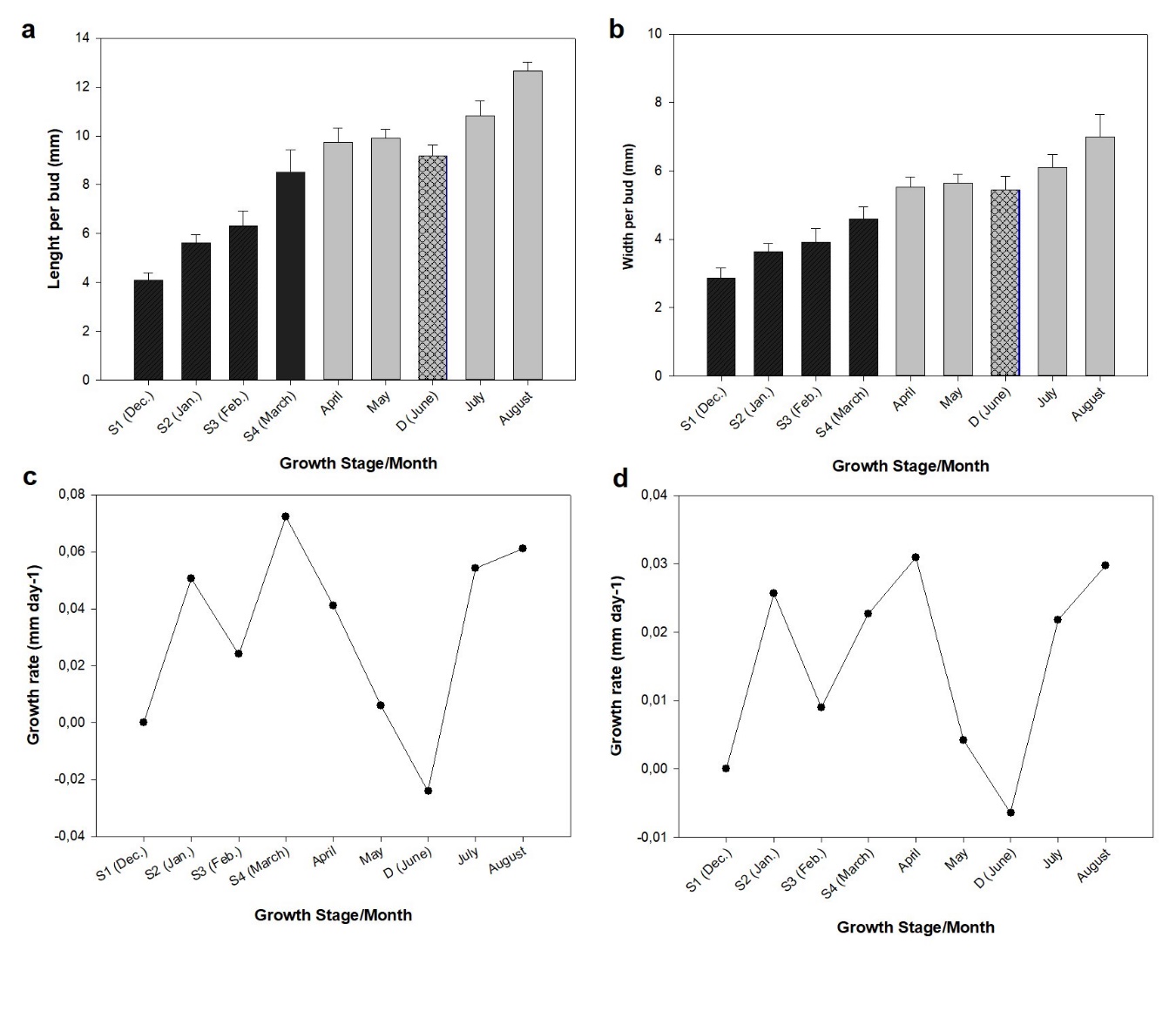
Fig S1: Bud growth and growth rates during floral bud differentiation stage (S1-S4) and dormancy (D) in sweet cherry (*P. avium* cv. Bing).** a, Length; b, width; c, growth rate of bud length; d, growth rate of bud width.
