# Supplementary material for: Comparative transcriptomic analysis reveals novel roles of transcription factors and hormones during the flowering induction and floral bud differentiation in sweet cherry trees (Prunus avium L. cv. Bing)
Source: PLoS One. 2020 Mar 12;15(3):e0230110. doi: 10.1371/journal.pone.0230110 (PMC7067470; doi:10.1371/journal.pone.0230110)
Supplement: S2 Fig — Samples were under five different developmental stages: S1 (flowering induction), S2, S3, S4 (flower whorls development) and D (dormancy). (DOCX) [file pone.0230110.s002.docx]

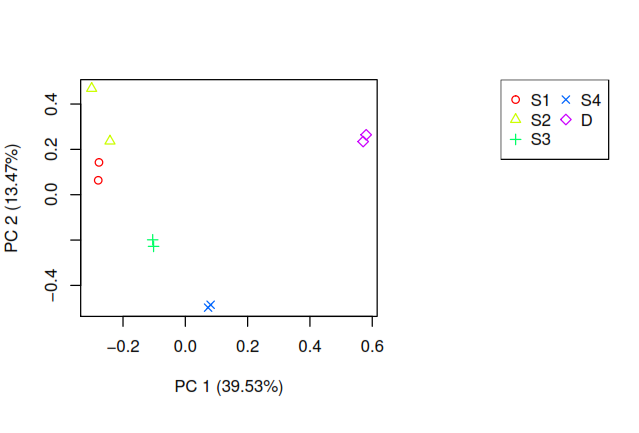


**Fig S2**: PCA analysis of the floral buds transcriptome data. Samples were under five different developmental stages: S1 (flowering induction), S2, S3, S4 (flower whorls development) and D (dormancy). Two biological replicates per sample are showed.
