# Supplementary material for: Comparative transcriptomic analysis reveals novel roles of transcription factors and hormones during the flowering induction and floral bud differentiation in sweet cherry trees (Prunus avium L. cv. Bing)
Source: PLoS One. 2020 Mar 12;15(3):e0230110. doi: 10.1371/journal.pone.0230110 (PMC7067470; doi:10.1371/journal.pone.0230110)
Supplement: S3 Fig — The DEGs from S1-S2, S2-S3 and S3-S4 floral bud transitions were compared. BP: Biological Process; CC: Cellular Component; MF: Molecular Function. (DOCX) [file pone.0230110.s003.docx]

**
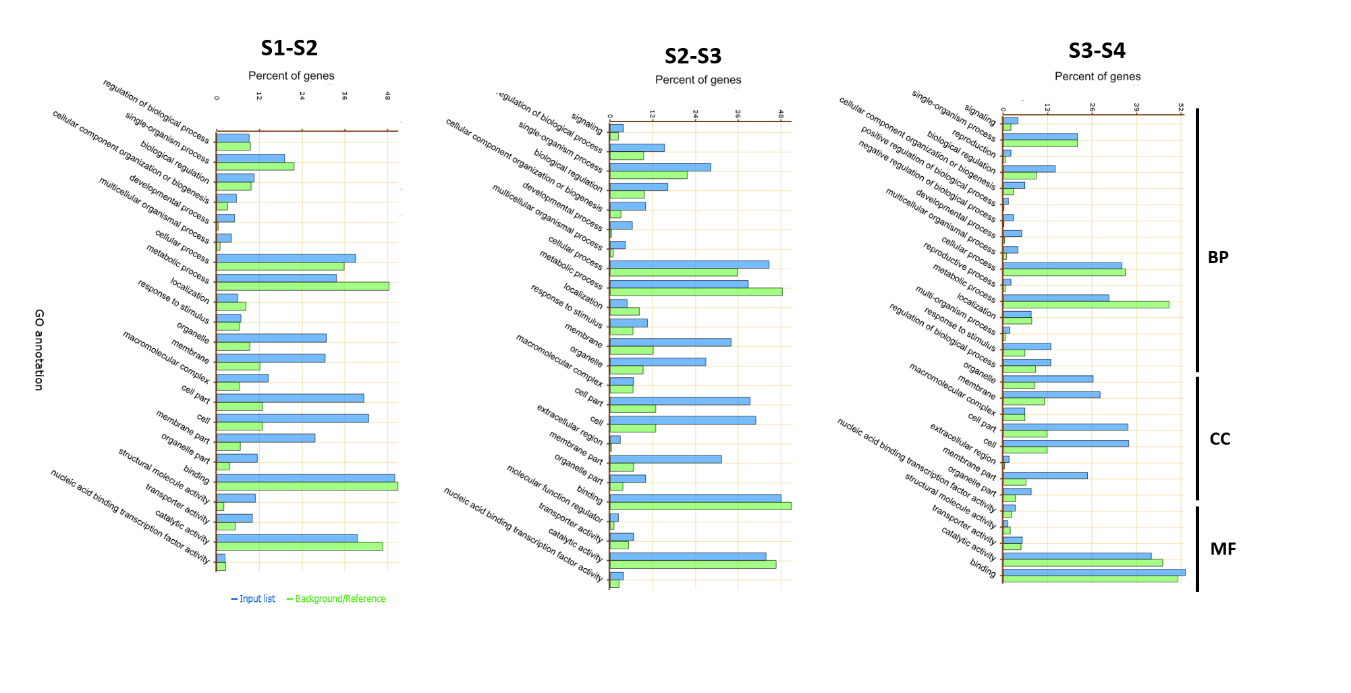
**

**Fig S3: Gene ontology term enrichment analysis based on SEA analysis of DEGs from floral buds**: the top enriched GO terms are listed. The DEGs from S1-S2, S2-S3 and S3-S4 floral bud transitions were compared. BP: Biological Process; CC: Cellular Component; MF: Molecular Function.
