# Supplementary material for: Comparative transcriptomic analysis reveals novel roles of transcription factors and hormones during the flowering induction and floral bud differentiation in sweet cherry trees (Prunus avium L. cv. Bing)
Source: PLoS One. 2020 Mar 12;15(3):e0230110. doi: 10.1371/journal.pone.0230110 (PMC7067470; doi:10.1371/journal.pone.0230110)
Supplement: S4 Fig — Expression patterns of selected differentially expressed transcription factors (TF) families in S1-S4 and D floral buds a) hierachical clustering of 24 transcripts codifyng bHLH TFs. b) hierachical clustering of 31 transcripts codifyng MYB TFs. c) hierachical clustering of 31 transcripts codifyng NAC TFs. The heat maps represent the log2 fold changes (FDR≤0.05) of DEGs TFs. Red and Green colors represent up- and down-regulated genes, respectively. Scale, representing the signal values, is shown at the top of the Fig. (DOCX) [file pone.0230110.s004.docx]

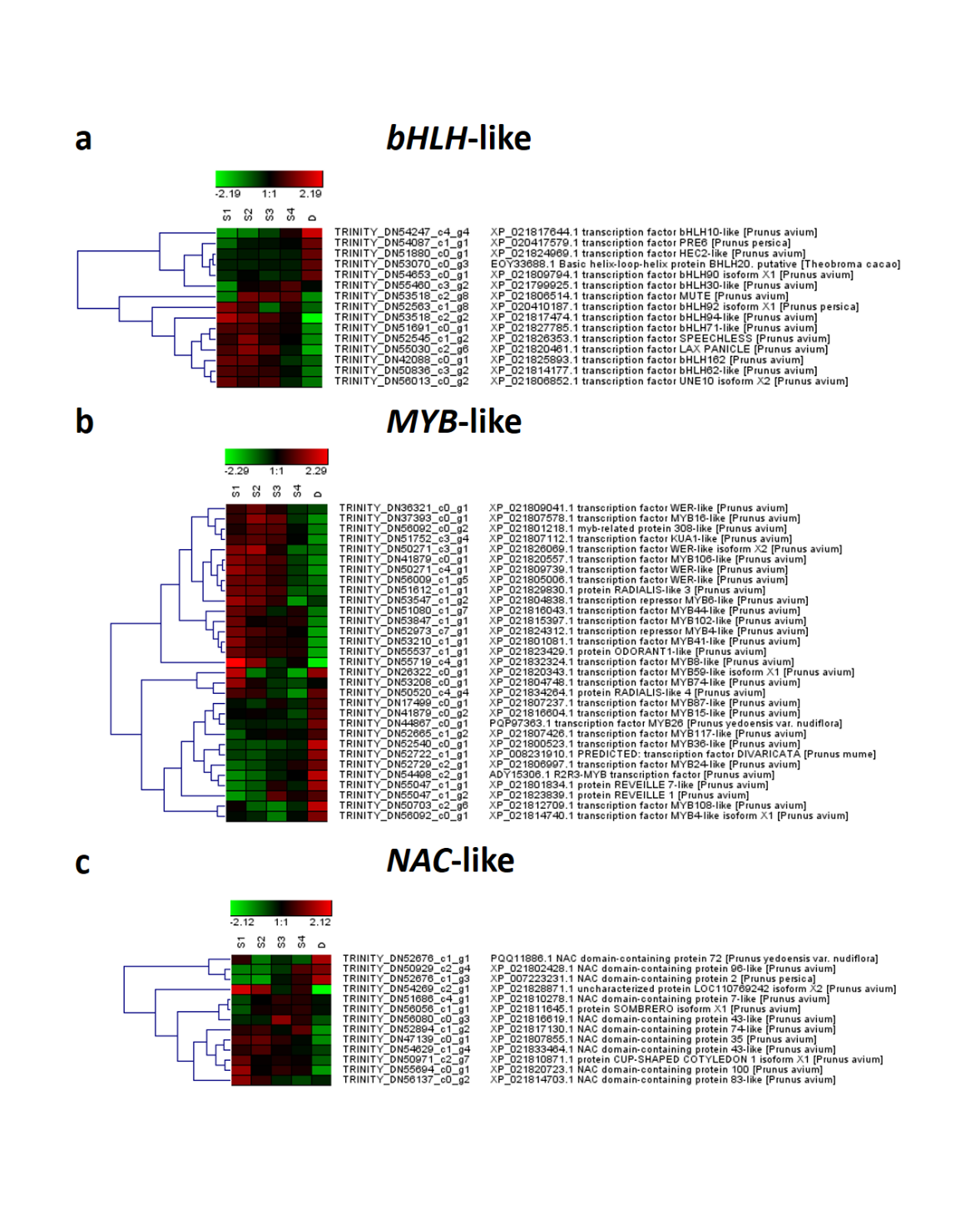


**Fig** **S4: Expression patterns of selected differentially expressed transcription factors (TF) families** **in S1-S4 and D floral buds** a) hierachical clustering of 15 DEGs codifyng bHLH TFs. b) hierachical clustering of 29 DEGs codifyng MYB TFs. c) hierachical clustering of 13 DEGs codifyng NAC TFs. The heat maps represent the log2 fold changes (FDR≤0.05) of DEGs TFs. Red and Green colors represent up- and down-regulated genes, respectively. Scale, representing the signal values, is shown at the top of the Fig.
