# Supplementary material for: Comparative transcriptomic analysis reveals novel roles of transcription factors and hormones during the flowering induction and floral bud differentiation in sweet cherry trees (Prunus avium L. cv. Bing)
Source: PLoS One. 2020 Mar 12;15(3):e0230110. doi: 10.1371/journal.pone.0230110 (PMC7067470; doi:10.1371/journal.pone.0230110)
Supplement: S5 Fig — The MADS-box subfamilies are indicated. The phylogram was generated with the MEGA 6.0 program from the multiple alignment of the deduced amino acid sequences from P. avium (Pav), Arabidopsis (At) and P. mume (Pm) MADS-box proteins. Bootstrap values from 1000 replicates were used to assess the robustness of the tree. Black and red dots indicate P. avium proteins. Also, red dots indicate P. avium genes analyzed by qPCR. (DOCX) [file pone.0230110.s005.docx]

**
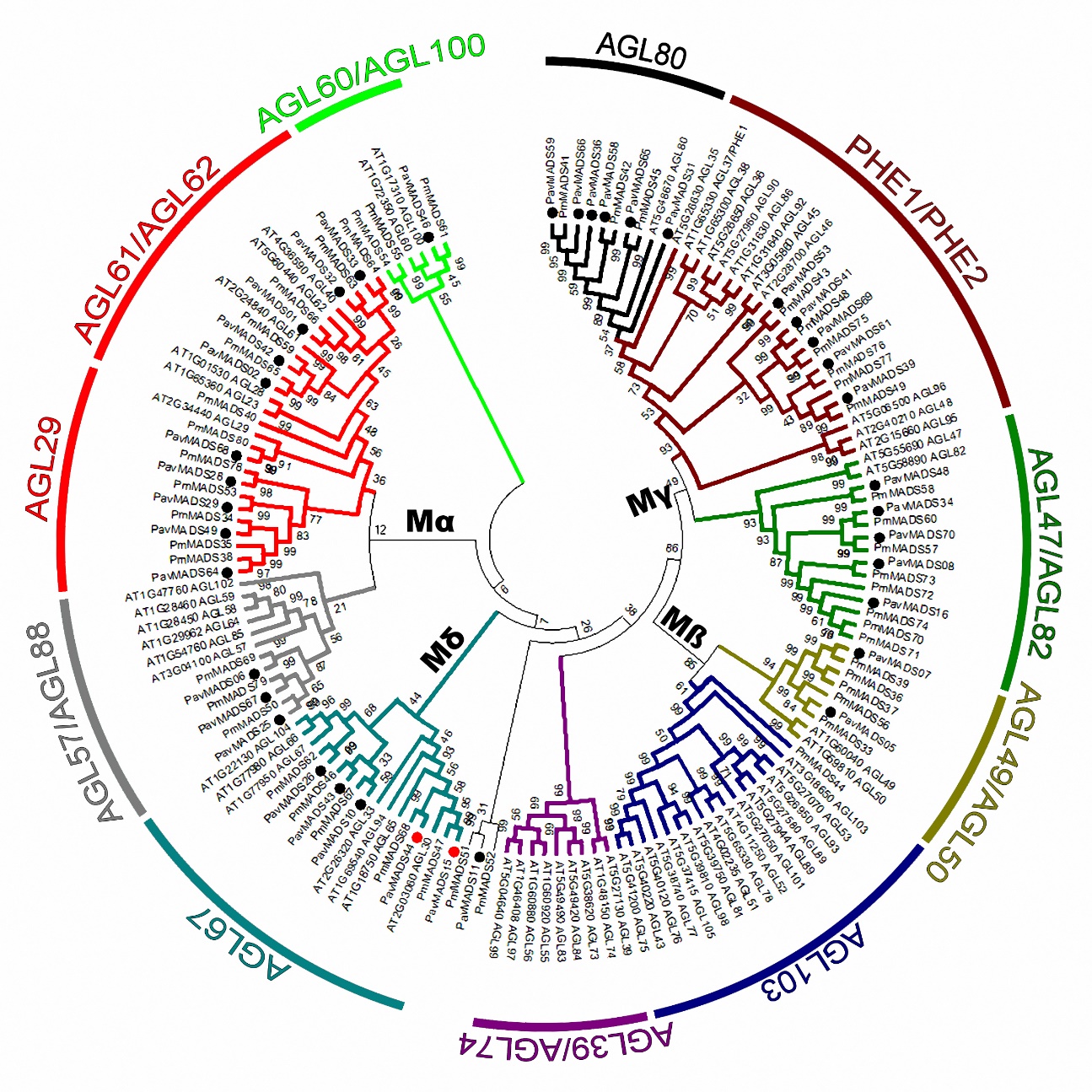
**

**Fig S5 Phylogenetic relationships among sweet cherry Type I MADS-box proteins.** The MADS-box subfamilies are indicated. The phylogram was generated with the MEGA 6.0 program from the multiple alignment of the deduced amino acid sequences from *P. avium* (Pav), *Arabidopsis* (At) and *P. mume* (Pm) MADS-box proteins. Bootstrap values from 1000 replicates were used to assess the robustness of the tree. Black and red dots indicate *P. avium* proteins. Also, red dots indicate *P. avium* genes analyzed by qPCR.
