# Supplementary material for: Comparative transcriptomic analysis reveals novel roles of transcription factors and hormones during the flowering induction and floral bud differentiation in sweet cherry trees (Prunus avium L. cv. Bing)
Source: PLoS One. 2020 Mar 12;15(3):e0230110. doi: 10.1371/journal.pone.0230110 (PMC7067470; doi:10.1371/journal.pone.0230110)
Supplement: S1 Table — (DOCX) [file pone.0230110.s007.docx]

**Table S1: Summary of transcriptomic sequencing of *P. avium* cv. Bing**
