# Supplementary material for: Comparative transcriptomic analysis reveals novel roles of transcription factors and hormones during the flowering induction and floral bud differentiation in sweet cherry trees (Prunus avium L. cv. Bing)
Source: PLoS One. 2020 Mar 12;15(3):e0230110. doi: 10.1371/journal.pone.0230110 (PMC7067470; doi:10.1371/journal.pone.0230110)
Supplement: S2 Table — (DOCX) [file pone.0230110.s008.docx]

**Table S2**: A catalog of *MADS*-box genes in sweet cherry

| **Gene name** | **GenBank accession** | **Protein length (aminoacids)** | **Subfamily** | **Family** |
| --- | --- | --- | --- | --- |
| PavMADS01 | XP_021800064.1 | 210 | AGL61/AGL62 | Mα |
| PavMADS02 | XP_021800067.1 | 240 | AGL61/AGL62 | Mα |
| PavMADS03 | XP_021800165.1 | 246 | AGAMOUS | MIKC^c^ |
| PavMADS04 | XP_021800912.1 | 203 | AGL17 | MIKC^c^ |
| PavMADS05 | XP_021802006.1 | 311 | AGL49/AGL50 | Mβ |
| PavMADS06 | XP_021802165.1 | 232 | AGL57/AGL88 | Mα |
| PavMADS07 | XP_021802701.1 | 319 | AGL49/AGL50 | Mβ |
| PavMADS08 | XP_021802934.1 | 154 | AGL47/AGL82 | Mγ |
| PavMADS09 | XP_021803407.1 | 221 | SOC1 | MIKC^c^ |
| PavMADS10 | XP_021804055.1 | 100 | AGL67 | Mδ |
| PavMADS11 | XP_021804143.1 | 199 | soloist | Mβ |
| PavMADS12 | XP_021806356.1 | 250 | AP1 | MIKC^c^ |
| PavMADS13 | XP_021806373.1 | 262 | SEPALLATA | MIKC^c^ |
| PavMADS14 | XP_021806588.1 | 210 | AP3/PI | MIKC^c^ |
| PavMADS15 | XP_021807370.1 | 250 | AGL67 | Mδ |
| PavMADS16 | XP_021807910.1 | 231 | AGL47/AGL82 | Mγ |
| PavMADS17 | XP_021808105.1 | 486 | SVP/DAM | MIKC^c^ |
| PavMADS18 | XP_021808108.1 | 445 | SVP/DAM | MIKC^c^ |
| PavMADS19 | XP_021808109.1 | 267 | SVP/DAM | MIKC^c^ |
| PavMADS20 | XP_021808113.1 | 242 | SVP/DAM | MIKC^c^ |
| PavMADS21 | XP_021808118.1 | 265 | SVP/DAM | MIKC^c^ |
| PavMADS22 | XP_021808126.1 | 239 | SVP/DAM | MIKC^c^ |
| PavMADS23 | XP_021809901.1 | 255 | AP1 | MIKC^c^ |
| PavMADS24 | XP_021809942.1 | 251 | SEPALLATA | MIKC^c^ |
| PavMADS25 | XP_021810048.1 | 211 | AGL57/AGL88 | Mα |
| PavMADS26 | XP_021810368.1 | 254 | AGL67 | Mδ |
| PavMADS27 | XP_021810915.1 | 228 | SVP | MIKC^c^ |
| PavMADS28 | XP_021811802.1 | 215 | AGL29 | Mα |
| PavMADS29 | XP_021812104.1 | 142 | AGL29 | Mα |
| PavMADS30 | XP_021813066.1 | 204 | AGL12 | MIKC^c^ |
| PavMADS31 | XP_021813095.1 | 159 | AGL80 | Mγ |
| PavMADS32 | XP_021814458.1 | 274 | AGL61/AGL62 | Mα |
| PavMADS33 | XP_021814539.1 | 274 | AGL61/AGL62 | Mα |
| PavMADS34 | XP_021814671.1 | 368 | AGL47/AGL82 | Mγ |
| PavMADS35 | XP_021814971.1 | 238 | AP3/PI | MIKC^c^ |
| PavMADS36 | XP_021815730.1 | 73 | AGL80 | Mγ |
| PavMADS37 | XP_021816776.1 | 268 | AP1 | MIKC^c^ |
| PavMADS38 | XP_021816782.1 | 246 | SEPALLATA | MIKC^c^ |
| PavMADS39 | XP_021817163.1 | 371 | PHE1/PHE2 | Mγ |
| PavMADS40 | XP_021818335.1 | 200 | TM8 | MIKC^c^ |
| PavMADS41 | XP_021818814.1 | 361 | PHE1/PHE2 | Mγ |
| PavMADS42 | XP_021819598.1 | 196 | AGL61/AGL62 | Mα |
| PavMADS43 | XP_021820249.1 | 392 | AGL67 | Mδ |
| PavMADS44 | XP_021820626.1 | 378 | AGL67 | Mδ |
| PavMADS45 | XP_021821687.1 | 254 | AGL15 | MIKC^c^ |
| PavMADS46 | XP_021822790.1 | 234 | AGL60/AGL100 | Mα |
| PavMADS47 | XP_021822939.1 | 215 | FLC | MIKC^c^ |
| PavMADS48 | XP_021823085.1 | 373 | AGL47/AGL82 | Mγ |
| PavMADS49 | XP_021824386.1 | 274 | AGL29 | Mα |
| PavMADS50 | XP_021825648.1 | 244 | AGAMOUS | MIKC^c^ |
| PavMADS51 | XP_021825726.1 | 244 | AGL6 | MIKC^c^ |
| PavMADS52 | XP_021825746.1 | 215 | SOC1 | MIKC^c^ |
| PavMADS53 | XP_021826211.1 | 224 | PHE1/PHE2 | Mγ |
| PavMADS54 | XP_021826391.1 | 262 | AGL15 | MIKC^c^ |
| PavMADS55 | XP_021826960.1 | 249 | AGL6 | MIKC^c^ |
| PavMADS56 | XP_021827090.1 | 213 | SOC1 | MIKC^c^ |
| PavMADS57 | XP_021829300.1 | 240 | SEPALLATA | MIKC^c^ |
| PavMADS58 | XP_021830441.1 | 243 | AGL80 | Mγ |
| PavMADS59 | XP_021830473.1 | 247 | AGL80 | Mγ |
| PavMADS60 | XP_021831173.1 | 322 | AGAMOUS | MIKC^c^ |
| PavMADS61 | XP_021831267.1 | 226 | PHE1/PHE2 | Mγ |
| PavMADS62 | XP_021831609.1 | 260 | AP3/PI | MIKC^c^ |
| PavMADS63 | XP_021832132.1 | 239 | AGL17 | MIKC^c^ |
| PavMADS64 | XP_021832457.1 | 209 | AGL29 | Mα |
| PavMADS65 | XP_021832692.1 | 229 | AGL80 | Mγ |
| PavMADS66 | XP_021833902.1 | 206 | AGL80 | Mγ |
| PavMADS67 | XP_021834216.1 | 209 | AGL57/AGL88 | Mα |
| PavMADS68 | XP_021834286.1 | 187 | AGL29 | Mα |
| PavMADS69 | XP_021834434.1 | 355 | PHE1/PHE2 | Mγ |
| PavMADS70 | XP_021834621.1 | 390 | AGL47/AGL82 | Mγ |
| PavMADS71 | XP_021834885.1 | 219 | SVP | MIKC^c^ |
