# Supplementary material for: Comparative transcriptomic analysis reveals novel roles of transcription factors and hormones during the flowering induction and floral bud differentiation in sweet cherry trees (Prunus avium L. cv. Bing)
Source: PLoS One. 2020 Mar 12;15(3):e0230110. doi: 10.1371/journal.pone.0230110 (PMC7067470; doi:10.1371/journal.pone.0230110)
Supplement: S4 Table — (DOCX) [file pone.0230110.s010.docx]

**Table S4: List of primers used in qPCR analysis of *MADS*-box genes**

| **Gene** | **Subfamily** | **Forwards (5’ a 3’)** | **Reverse (5’ a 3’)** |
| --- | --- | --- | --- |
| *PavMADS03* | AG/SHP | CAGTCAATGCCTTCACAGTCA | GGATAGAATGGAACATCAGACG |
| *PavMADS14* | AP3/PI | ACACGAAAGGCAAACGGTATCTGG | AAGTAACCGGGACAAGCAGTCCAA |
| *PavMADS15* | AGL30 | TGCTGTTACCATGTTTGACGA | CCACGGCAAAAATTACGATT |
| *PavMADS21* | SVP/DAM1 | GAGCCGGAGAGGTTGAATAA | CACACAATCACAACCCTCCA |
| *PavMADS27* | SVP | TTGCAGAAGAAGCAGAAGCA | GGAGCTGGCGTATTCAAAGA |
| *PavMADS38* | SEP | TGTGGTTGATTGGGTGGATA | GCTTGCCACGGTTAGAGAAG |
| *PavMADS40* | TM8 | CATTGCAAGAAGAAAACAGTCG | AAGTACTTGGTGCGAGAGATCA |
| *PavMADS44* | AGL65 | CCGTCGTGTGCAAATCTAAA | CGGTTACCACTGCTCTCCA |
| *PavMADS45* | AGL15 | AGAGACCGAGACAGAGAAAACC | AATCTTCCCCCTACCCATCT |
| *PavMADS51* | AGL6 | GAAGAGCATGGCTTGTGAGA | CCATAGGGCCATATATTCTTGC |
| *PavMADS54* | SEP | AAGCATTTTCAGTGGTTTCCA | CTTGAGCAGCCCATTTCTTC |
| *PavMADS57* | SEP | AGCAGGTCCAAGCGTGAGTA | GCACCCACAACTCTTCAGGT |
